# Assessing the role of T cells in response to retinal injury to uncover new therapeutic targets for the treatment of retinal degeneration

**DOI:** 10.1186/s12974-023-02867-x

**Published:** 2023-09-09

**Authors:** Federica M. Conedera, Judith M. Runnels, Jens V. Stein, Clemens Alt, Volker Enzmann, Charles P. Lin

**Affiliations:** 1 Department of Oncology, Microbiology and Immunology, University of Fribourg, Fribourg, Switzerland; 2 Department of Ophthalmology, Bern University Hospital, Bern, Switzerland; 3 Department of BioMedical Research, University of Bern, Bern, Switzerland; 4 Center for Systems Biology and Wellman Center for Photomedicine, Massachusetts General Hospital and Harvard Medical School, Boston, MA USA

**Keywords:** Retinal laser-injury, Mouse, Human, Microglia, CD8^+^ T cells, Adaptive immune cells, In vivo imaging

## Abstract

**Background:**

Retinal degeneration is a disease affecting the eye, which is an immune-privileged site because of its anatomical and physiological properties. Alterations in retinal homeostasis—because of injury, disease, or aging—initiate inflammatory cascades, where peripheral leukocytes (PL) infiltrate the parenchyma, leading to retinal degeneration. So far, research on PL's role in retinal degeneration was limited to observing a few cell types at specific times or sectioning the tissue. This restricted our understanding of immune cell interactions and response duration.

**Methods:**

In vivo microscopy in preclinical mouse models can overcome these limitations enabling the spatio-temporal characterization of PL dynamics. Through in vivo imaging, we assessed structural and fluorescence changes in response to a focal injury at a defined location over time. We also utilized minimally invasive techniques, pharmacological interventions, and knockout (KO) mice to determine the role of PL in local inflammation. Furthermore, we investigated PL abundance and localization during retinal degeneration in human eyes by histological analysis to assess to which extent our preclinical study translates to human retinal degeneration.

**Results:**

We demonstrate that PL, especially T cells, play a detrimental role during retinal injury response. In mice, we observed the recruitment of helper and cytotoxic T cells in the parenchyma post-injury, and T cells also resided in the macula and peripheral retina in pathological conditions in humans. Additionally, we found that the pharmacological PL reduction and genetic depletion of T-cells reduced injured areas in murine retinas and rescued the blood–retina barrier (BRB) integrity. Both conditions promoted morphological changes of Cx3cr1^+^ cells, including microglial cells, toward an amoeboid phenotype during injury response. Interestingly, selective depletion of CD8^+^ T cells accelerated recovery of the BRB compared to broader depletions. After anti-CD8 treatment, the retinal function improved, concomitant to a beneficial immune response.

**Conclusions:**

Our data provide novel insights into the adaptive immune response to retinal injury in mice and human retinal degeneration. Such information is fundamental to understanding retinal disorders and developing therapeutics to modulate immune responses to retinal degeneration safely.

**Supplementary Information:**

The online version contains supplementary material available at 10.1186/s12974-023-02867-x.

## Introduction

Despite considerable effort and significant therapeutic advances, retinal degenerative diseases remain the most common cause of blindness in the developed world. While treatments are available to manage late-stage symptoms (e.g., VEGF inhibitors [1], corticosteroids [2], and photodynamic therapy [3]), retinal degeneration has no proven treatment. Recently, there has been significant interest in the possibility that cellular therapies, such as immunomodulation, may slow or reverse vision loss [4].

The immune system is divided into two distinct parts: innate and adaptive [5]. The innate immune system includes monocytes, macrophages and neutrophils, and is tasked with delivering host defense rapidly after tissue insult. The adaptive immune system includes T cells, and their exposure to pathogenic antigens confers long-term defense memory in the host organism [6]. In healthy conditions, the blood–retina barrier (BRB) excludes peripheral innate and adaptive immune cells from the retinal tissue. Meanwhile, the local immune response is provided by resident immune sentinels, known as microglia. During degeneration, circulating innate and adaptive immune cells can invade the retina because the BRB is compromised [7].

Whereas interactions between innate immune cells and microglia during retinal degeneration have been widely explored, the role of T cells has so far received little attention [8]. Nevertheless, both types of T cells, cytotoxic (expressing cluster of differentiation 8, CD8^+^) and helper (expressing cluster of differentiation 4, CD4^+^), are associated with retinal degeneration [9]. Whether T cell responses relay pathogenic or beneficial functions remains unclear; this might be due to the diversity of T-cell subsets and their systemic and local modes of action [10]. Circulating CD8^+^ T cells antagonize the function of protective immune cells [11], while locally limiting persistent inflammation [12, 13]. Furthermore, a subpopulation of CD4^+^ regulatory T cells (expressing forkhead box P3, Foxp3^+^) affects the local inflammatory response in the retina [14]. However, their differentiation from circulating lymphocytes cannot occur during retinal degeneration due to systemic, low-grade inflammation by circulating T cells [15]. Although early evidence indicates that CD4^+^ and CD8^+^ T cell populations are related to the pathophysiology of retinal degenerations, their interactions and activation mechanisms are still unclear. There is a compelling need to understand the specific role of T-cells in response to induced retinal degeneration and how their modulation governs complex cell–cell interactions that, in turn, may influence the ability of the retina to recover from injury.

Here, we used a scanning laser ophthalmoscope (SLO) to longitudinally image the murine retina during the response to focal laser-injury to define the role of T cell subsets on retinal degeneration. Our custom-built SLO is a confocal microscope that uniquely enables in vivo multi-color imaging of the murine retina. Furthermore, a solid-state laser (532 nm) and an acousto-optic modulator (AOM) are embedded in the SLO to induce a focal injury to the retina under image guidance.

To this end, we combined in vivo imaging, pharmacological interventions, and knockout (KO) mouse models to determine the role of CD4^+^ and CD8^+^ T cells during the local inflammatory response associated with tissue pathophysiology. Furthermore, we investigated the abundance and localization of T cells during retinal degeneration in humans by post-mortem tissue analysis, allowing us to assess to which extent our preclinical study in mice translates to human retinal degeneration. Such information is fundamental to understanding retinal disorders and developing targeted therapeutic strategies that can safely modulate the immune response to retinal degeneration and improve outcomes.

## Methods

### Animals

All experimental protocols were approved by the Institutional Animal Care and Use Committee of Massachusetts General Hospital and the governmental authorites of the Canton of Bern, Switzerland. Male and female 12-week-old C57Bl/6J mice were purchased from Jackson Laboratories (Bar Harbor, ME, USA). Homozygous Cx3cr1^GFP^Ccr2^RFP^ mice (B6.129(Cg)-Cx3cr1tm1Litt Ccr2tm2.1Ifc/JernJ) were obtained from Jackson Laboratory (strain 032127) and crossed with C57Bl/6J mice to obtain heterozygotes. Cx3cr1^GFP^Ccr2^RFP^ animals express green fluorescent protein (GFP^+^) in resident immune cells, such as microglia, and blood-borne monocytes/macrophages [16–18] and red fluorescent protein (RFP^+^) in peripheral leukocytes (PL; [19, 20]). PL expressing Ccr2 include monocytes/macrophages, T cells and neutrophils, as reported in the literature [19, 21–24]. Since blood-borne monocytes/macrophages can be both Cx3cr1^+^ and Ccr2^+^ and thus RPF^+^GFP^+^, resident immune cells were distinguished as Cx3cr1^+^Ccr2^−^ and therefore only GFP^+^ [25].

Thus, they were employed to evaluate in vivo the immune response to retinal injury. Although there are reports of sex differences in immune reactivity [26], no differences were observed during imaging experiments; thus, both male and female animals were used, and the results were combined. Foxp3^GFP^ knock-in mice (C57B/6L background) were provided by Terry Strom's laboratory. Rag1 KO mice (B6.129S7-Rag1tm1Mom/J), in which lymphocytes are absent in the whole body, were also purchased from Jackson Laboratory and maintained as homozygotes (strain 002216). Homozygous mice deficient in perforin (Prf1 KO mice, C57BL/6-Prf1tm1Sdz/J) were obtained from Jackson Laboratory and maintained as homozygotes (strain 002407). All animals were housed in designated animal holding facilities observing a standard 12-h day/night cycle. Standard rodent chow and water were provided ad libitum.

### In vivo retinal imaging

Our SLO has been set up for multi-color confocal imaging of the murine retina [27, 28]. A diode laser with 638 nm wavelength (Micro Laser Systems, Inc. Garden Grove, CA, USA) was used to detect reflected light and excite AlexaFluor647 (AF647). A two-color laser (Dual Calypso, Cobolt AB, Vretenvägen, Sweden) excites GFP or fluorescein with 491 nm and RFP with 532 nm. A spinning polygon scanner (Lincoln Laser Corp., Phoenix, AZ, USA) and a galvanometric mirror (GSI Lumonics, Billerica, MA, USA) raster scanned a field of view of 425 to 575 µm on the retina at 30 frames per second. The laser power incident on the cornea was 0.6 mW for the 638 nm diode laser, 0.5 mW for the 532 nm laser, and 0.5 mW laser power for the 491 nm laser. Reflectance images were acquired by separating the backscattered from the vertically polarized incident light by means of a quarter-wave plate and a polarizing beam splitter cube. Light reflected from the retina is horizontally polarized after the double-pass through the quarter-wave plate and is reflected by the polarizing beam splitter, focused through a confocal pinhole (diameter 25 µm ≈ 1.25 airy disc size) and detected by a photomultiplier tube (PMT; R3896, Hamamatsu, Japan). Fluorescence from the retina was transmitted into a dedicated fluorescence detection arm through a dichroic beam splitter (Di03-R405/488/532/638, Semrock, Rochester, NY, USA), separated into three distinct channels by 560 nm and 650 long-pass dichroic beam splitters, and detected through a 650 nm long-pass filter (AF647, Evan Blue), 525/50 (GFP), and 593/40 (RFP) bandpass filters (all Semrock). All fluorescence channels detected light through a confocal pinhole (diameter 50 µm ≈ 2.5 airy disc sizes) with a PMT (R3896, Hamamatsu).

Mice were placed in a heated holder integrated with a nose cone for inhalation anesthesia (1–2% isoflurane in oxygen). Their pupils were dilated with Tropicamide 1% Ophthalmic solution (Bausch & Lomb Inc, Tampa, FL, USA). A contact lens (diameter 2.5 mm, base curvature 1.65 mm, power: 12D, material PMMA; Unicon Corporation, Osaka, Japan) was placed on the mydriatic eye, and a drop of GenTeal eye gel (Alcon, Fort Worth, TX, USA) prevented the cornea from drying out. In vivo images were recorded before injury (baseline), immediately after and 1, 4, 7, 10 and 14 days after the generation of the damage. After each imaging session, mice were returned to their cages where they became fully ambulatory within ∼10 min.

### Retinal laser-injury

For assessment of cellular retinal response, a coagulator was embedded in the SLO to allow image-guided retinal coagulation [28]. It consists of a high-power continuous-wave (CW) laser (Ventus, Laser Quantum, Cheshire, UK) and an acousto-optic modulator (AOM; TEM-85-1-.532, Brimrose, Baltimore, MD, USA) that allows pulses to be chopped from the laser emission. A small mirror reflected the 0-order beam of the AOM into a beam dump; the first-order beam was directed onto a tip-tilt-scanner. The scanner was placed in a plane conjugate with the mouse eye pupil through a telecentric relay system of two 75 mm focal length lenses. The scanner served to position the coagulator spot onto a retinal parenchyma. The coagulation beam was combined with the SLO excitation lasers by a removable dichroic beam splitter (DiO1-R532, Semrock). This beam splitter allows placing of 532 nm laser pulses into the retinal parenchyma under the guidance of simultaneous real-time imaging by the SLO using the 638 nm laser. The laser beam had a diameter of 100 µm in the the retina. Anesthetized mice received a single laser burn in the nasal region of the retina at a distance of at least one lesion diameter from the ONH with a 25 ms laser pulse of 75 mW power to minimize collateral damage to surrounding tissue.

For electroretinography (ERG) measurements, a different 532 nm diode laser (Visulas 532s, Carl Zeiss Meditec AG, Oberkochen, Germany) was used to rapidly generate up to 50 lesions at the posterior pole region around the optic nerve head (ONH; [29, 30]). Mice were anesthetized by injecting subcutaneously 45 mg/kg ketamine (Ketalar 50 mg/ml; Orion Pharma AG, Zug, Switzerland) and 0.75 mg/kg medetomidine hydrochloride (Domitor, 1 mg/ml; Orion Pharma AG). Pupils were dilated using tropicamide 0.5% and phenylephrine HCl 2.5% (MIX-Augentropfen; ISPI, Bern, Switzerland). Afterward, a few drops of 2% hydroxypropyl methylcellulose (OmniVision, Neuhausen, Switzerland) were applied topically to the cornea. To focus, the laser-aiming beam on the retina a 2.0-mm laser lens was employed (Ocular Instruments, Bellevue, WA, USA). We generated as many laser burns as possible in both eyes (50 injuries/eye), maintaining a space of at least one spot size between each laser burn. Each burn was produced with 120 mW of power for 60 ms and 100 μm in diameter.

### Fluorescein angiography (FA)

To visualize the BRB leakage in vivo, we injected 50 μL of 0.01% fluorescein (AK-Fluor® 10%; Akorn Inc., Gurnee, IL, USA) retro-orbitally. Because of the high permeability of the BRB during degeneration, it is necessary to perform dye injection while the mouse is on stage so that the early dynamics of dye leakage immediately after injection can be observed. Videos (10 s) recorded the reflectance and fluorescence channels simultaneously every 30 s over 5 min. We performed FA before (baseline) and after laser-injury at different time points (days 0, 1, 4, 7, 10 and 14).

### Labeling of T cells for in vivo imaging

C57Bl/6J mice were injected retro-orbitally with 5 µg of AlexaFluor488 (AF488) anti-CD4 antibody (100529; Biolegend, San Diego, CA, USA) and 5 µg AF647 anti-CD8 antibody (100724; Biolegend) 40 min before imaging. Antibodies were injected on days 1, 3, 7, 10, and 14 post-injury for longitudinal imaging of the adaptive immune response. Repeated doses of anti-CD4 or -CD8 at a low concentration avoid cell depletion, such that T cells could be visualized for 8–10 h. Additionally, blood was collected from the submandibular vein 40 min after injection to validate the accuracy of antibody labeling by flow cytometry (Additional file 1: Fig. S1). Tracers were detected by acquiring still images. A time-lapse image sequence was obtained by taking one image every 30 s over 5 min. The time-lapse stack was aligned to correct motion artifacts. Then an average image was generated from the time-lapse stack to reduce image noise. The presented images were contrast stretched to the same white and black levels for display purposes. All image processing was undertaken in ImageJ.

### Pharmacological treatments

FTY720 (fingolimod) is an immunosuppressive compound based on its effects on leukocyte migration via the signaling of sphingosine-1-phosphate receptor 1 (S1p1; [31]). The compound was dissolved in water (1.85 mg/L) and supplied ad libitum to C57Bl/6 J or Cx3cr1^GFP^Ccr2^RFP^ mice every 5 days starting 5 days before injury induction (estimated dosage of 1.25 mg/kg/day; [32]). The control group received normal water, and mice thus were considered untreated. Blood was collected from the submandibular vein 24 h before injury and a week after (day 7) to monitor the extent of depletion by flow cytometry. Blood samples of approximately 100 μl volume were collected in heparin and incubated for 15 min in 3 ml of red blood cell lysis buffer (11814389001; Roche, Indianapolis, IN, USA) to remove RBCs. Afterward, 10 ml of cold PBS with 2% fetal bovine serum was added to the solution. This step was repeated after centrifugation at 400 × g for 5 min. Samples were stained with the following fluorescently labeled markers: Zombie Aqua™ Fixable Viability Kit (423101; Biolegend), FITC anti-mouse CD45 antibody (103108; Biolegend), APC/Cyanine7 anti-mouse CD3 antibody (100222; Biolegend), and PE anti-mouse/human CD45R/B220 antibody (103208; Biolegend). Samples were resuspended in PBS with 2% FBS alone and then analyzed the same day using the BD FACSAria™ II Cell Sorter. Furthermore, in vivo imaging was used to verify the absence of CD4^+^ and CD8^+^ cell infiltration in the retina.

Mice were depleted of either CD4^+^ or CD8^+^ T cells, as previously described [33]. Neutralizing monoclonal antibody CD4 GK1.5 or CD8 2.43 1 mg/kg (BE0003-1 and BE0061; BioXCell, Lebanon, PA, USA) were diluted in PBS and injected intraperitoneally into C57Bl/6J or Cx3cr1^GFP^Ccr2^RFP^ mice for three consecutive days before laser-injury, and weekly following injury (day 8). The control group of each antibody treatment received an injection of the vehicle (PBS) or a sham injection; thus, mice were considered untreated. We performed flow cytometry after CD4 and CD8 depletion, demonstrating the effective depletion of the T-cell subset of interest (Additional file 2: Fig. S2a, b). Blood was collected from the submandibular vein of either Cx3cr1^GFP^Ccr2^RFP^ or C57Bl/6J mice 24 h before and after injury.

### Spectral domain-optical coherence tomography (SD-OCT)

In vivo cross-sectional images of the murine retina were acquired with a Heidelberg Spectralis SLO and OCT combination (Spectralis HRA + OCT; Heidelberg Engineering GmbH, Heidelberg, Germany) as previously described [30, 34]. After anesthesia (see above), we dilated the pupils with a solution of tropicamide 0.5% and phenylephrine 2.5% (ISPI), and we hydrated the eyes with methylcellulose (OmniVision) before each imaging. SD-OCT images were obtained from both eyes using a 55° lens at a high resolution of 1008 × 596 pixels in grid mode. After centering on the ONH, 25 to 50 images were taken, and representative scans were selected for the final pictures. After the SD-OCT session, the anesthesia was reversed by injecting 2.5 mg/kg atipamezole (Antisedan 5 mg/ml; Provet AG, Lyssach, Switzerland). To quantify the damage inflicted on each mouse, ten measurements were taken per animal and then averaged. The injured area was determined by fitting an oval shape to each injury and measuring the semi-major and semi-minor axis length of an ellipse using the Heidelberg Eye Explorer software (Heidelberg Engineering GmbH) if it was visible in the retinal fundus.

### Electrophysiological test

Mice were dark-adapted overnight in dim red light. Before each ERG recording, the animals were anesthetized as described above, and pupils were dilated at the time of anesthesia by topical instillation of 2.5% phenylephrine and 0.5% tropicamide (ISPI). Oxybuprocaine (Oxybuprocaine 0.4% SDU Faure; Thea Pharma, Schaffhausen, Switzerland) was used for additional local anesthesia. Reference electrodes were inserted 1 cm under each ear towards 1 mm under the eye, a ground electrode was inserted subcutaneously at the tail base, and recording gold electrodes were placed onto mouse corneas. As previously reported [35], the anesthetized mice were placed into the recording apparatus on a temperature-controlled heating table (Ganzfeld stimulator Q400; Roland Consult, Brandenburg, Germany). The scotopic ERG was performed at 10 different white-flash stimuli ranging from − 40 dB (0.00030 cd*s/m^2^) to 15 dB (30 cd*s/m^2^). Subsequently, light adaptation was accomplished with low background illumination starting 10 min before photopic recording, and the eyes were subjected to flashes of 8 different light intensities from − 20 dB (0.030 cd*s/m^2^) to 15 dB (30 cd*s/m^2^). Ten flashes per light intensity (256-ms duration) were presented at 0.2 Hz with a 20-ms interval, and the responses were averaged. A- and b-wave amplitudes were quantified using the RETI-port/scan 21 analysis tool (Roland Consult), and the anesthesia was revoked by injection of 2.5 mg/kg atipamezole (Antisedan; Orion Pharma).

Data recording and analysis were performed using the RETI-port software (Roland Consult). The a-wave, characterized by its initial negative deflection lasting for approximately 20 ms, was followed by the subsequent positive deflection referred to as the b-wave, which persisted for approximately 65 ms. The RETI-port software automatically detects both a- and b-waves, quantifying the a-wave from baseline to the bottom of the a-wave trough, and the b-wave amplitude was measured from the a-wave trough to the b-wave peak.

### Immunofluorescence

First, we tested the presence of T cells in murine retinas. Mice were euthanized, and their eyes were enucleated 4 days after laser induction. The specimens were fixed overnight in 4% paraformaldehyde (PFA) in PBS. Paraffin sections (5 μm) were utilized for immunofluorescence. Antigen retrieval was achieved by incubating the sections in citrate buffer (pH 6.0) with 0.05% Tween-20 for 20 min, then cooling at room temperature for approximately 30 min. Blocking was performed for 1 h in Tris-buffered saline (TBS) containing 5% goat normal serum and 1% bovine serum albumin (pH 7.6). The sections were then incubated overnight at 4 °C with a rabbit anti-mouse CD3 antibody (ab237721, Abcam, Cambridge, UK). After washing with PBS and 0.05% Triton X-100, the sections were incubated with secondary antibodies conjugated to 594 fluorophores (AF488594 Abcam). Cell nuclei were counterstained using mounting media Vectashield with 4′,6-diamidino-2-phenylindole (DAPI; Vector Labs, Burlingame, CA, USA). Additionally, the study was carried out on paraffin sections from human eye donors (70–90 years old). As drusen are an early pathological feature of retinal degeneration, retinal specimens were divided based on their presence or absence between the retinal pigment epithelium (RPE) and Bruch’s membrane, as previously reported [29]. We examined retinas from 16 donors, 8 of which were healthy retinas and 8 were drusen-positive retinas (Drusen) with either micro-drusen singly or in a row (< 25 μm) or hyalinized round deposits (> 25 μm). From each donor, both macula and retinal periphery were analyzed. Samples were obtained from donors' eyes after the removal of the cornea for transplantation. Eyes from donors suffering from systemic or ocular comorbidities were excluded based on the criteria for the corneal donations (e.g., sepsis, meningitis, HIV, lues, hematological neoplasms, all ocular tumors, Creutzfeldt–Jakob disease, rapid progressive dementia or degenerative neurological condition, eye surgery within 6 months or after transplantations, drug abuses). The research complies with the Swiss human research act.

Within 24 h post-mortem, retinal samples were fixed with 4% formaldehyde at 4 °C overnight and embedded in paraffin. Thin sections (5 µm) of the posterior segment were cut, and the area of interest was selected based on the distance from the macula in the center of the macula identified as a depression in the retinal cross-section. The periphery was defined as sections 500 µm (100 cuts) away from the macula. Antigen retrieval was achieved by incubation in either Tris–EDTA (pH 9.0) or citrate buffer (pH 6.0) with 0.05% Tween-20 for 20 min and then cooled at room temperature (~ 30 min). All sections were blocked for 1 h in Tris-buffered saline + 5% goat normal serum + 1% bovine serum albumin (pH 7.6) and incubated with primary antibodies overnight at 4 °C. Primary antibodies used in this study were mouse α-human CD45 (555480BD; Pharmingen, BD Biosciences, Franklin Lakes, NJ, USA) and rat anti-human CD3 antibodies (MCA1477T; Bio-Rad, Hercules, CA, USA). This step was followed by washing with PBS and 0.05% Triton X-100 and incubation with the respective secondary antibodies conjugated to 488/594 fluorophores (AF488 and 594 Abcam). Cell nuclei were counterstained using the mounting media Vectashield with DAPI (Vector Labs).

### Quantification and statistical analysis

The analyzed area of the murine retina was confined to the injury detected in reflectance. The damaged site was identified as a hyper-reflective tissue (10% higher intensity compared to healthy parenchyma) outlined by a hypo-reflective circle (10% lower intensity compared to healthy parenchyma). The number of positive cells visible during in vivo imaging, the injury and FA area from in vivo experiments, and immunofluorescence were manually determined with ImageJ [36]. Representative figures show the segmentation process for identifying Cx3cr1^+^, Ccr2^+^, CD4^+^, CD8^+^, and Foxp3^+^ cells from pictures obtained by in vivo imaging (Additional file 3: Fig. S3). However, additional studies will be needed to validate the actual cell numbers in the retina. Furthermore, Cx3cr1^+^ cell polarization toward the damage site was measured by adapting the formula proposed by Lee et al. [37]. A polarization coefficient, *P*, was defined as: *P* = [AVG(Dp—Ds)]/Kd, where Dp = distance of process tip from the center of laser-injury, Ds = distance of soma from the damage site, and Kd = average diameter of Cx3cr1^+^ soma. We quantified the polarization coefficient of Cx3cr1^+^ cells within the field of view (≈425 µm) and then averaged our measurements per mouse. Furthermore, each time point (days 1, 4, 7, 10, 14) was normalized to the baseline (before injury) or day 0 (immediately after injury). Quantitative analysis of primary and terminal processes of Cx3cr1^+^ cells were restricted to Cx3cr1^+^ cells within the field of view, avoiding changed-focus artifacts as it might cause misleading structural changes. Spearman's correlation matrix was generated with R v. 3.5.1 using the corrplot function from package gplots v. 3.0.1.

All measurements were performed blinded to the treatment group, and normality tests were performed before statistical tests, where suitable. When normality was met, parametric analysis (*t*-test or one-way ANOVA with Tukey’s or Bonferroni post-test to account for multiple comparisons) was performed, and means were reported. Otherwise, non-parametric analyses (Mann–Whitney test, Spearman rank-sum correlation) were performed, and the median values were reported. For longitudinal measurements, we used linear mixed-effects modeling. The specific test used is indicated in the text and figure legends. Differences were considered statistically significant at the *p*-value ≤ 0.05. All statistical analyses were performed in GraphPad Prism 9.4.1 (GraphPad Software, Boston, MA, USA).

## Results

### Impact of leukocyte infiltration on inflammatory response and tissue repair after laser-induced injury

**Fig. 1:**
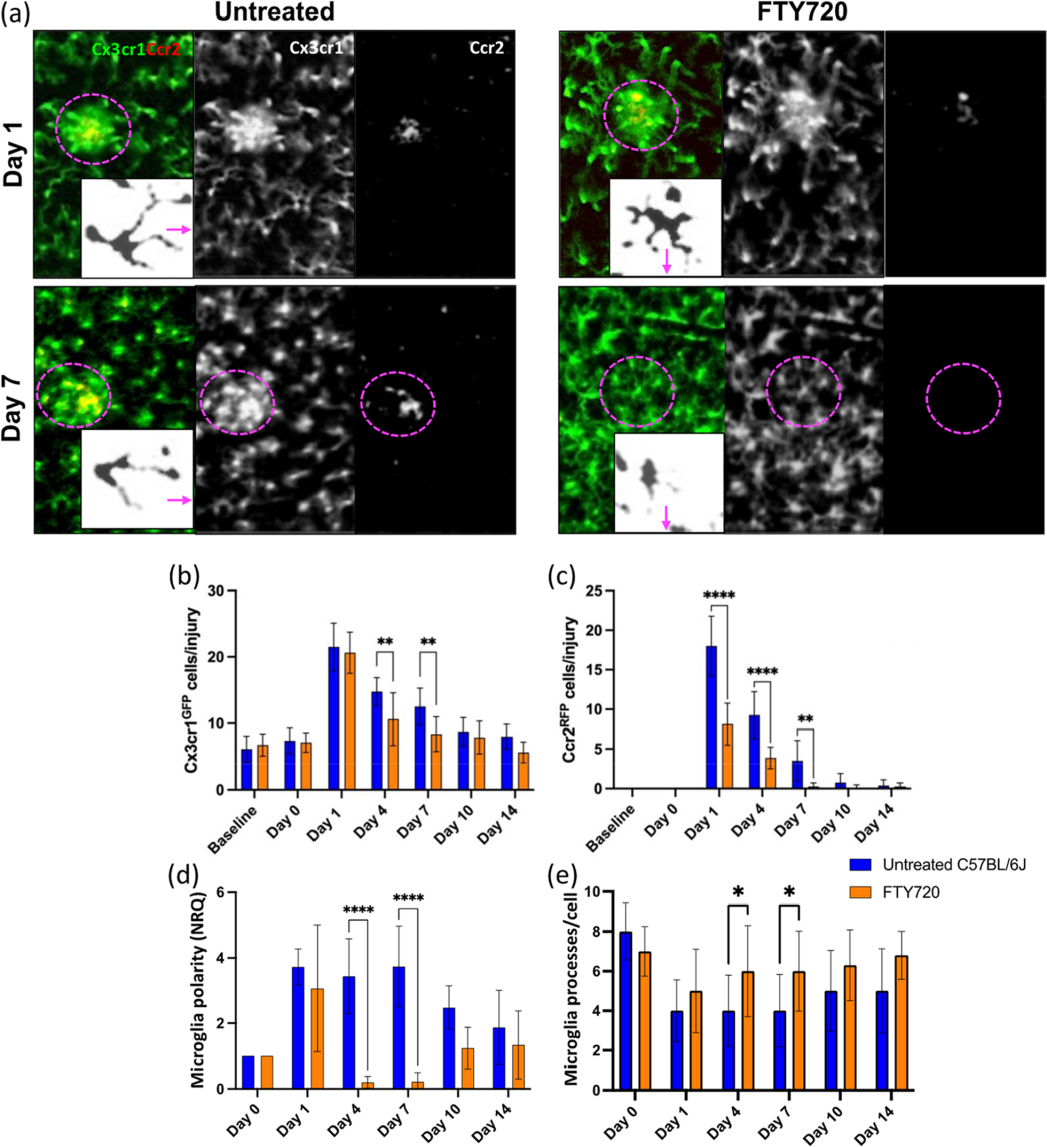
Repeated FTY720 dose regimen reduces retinal inflammatory response to laser-induced injury. **a**–**d** Inflammatory response of untreated and FTY720-treated Cx3cr1^GFP^Ccr2^RFP^ mice. **a** Images show differences in resident and blood-borne immune cell recruitment on days 1 and 7 in the damaged site (delimited by magenta dashes). By day seven post-injury, untreated mice still exhibit clustered Cx3cr1^+^ cells and PL at the site of damage a week post-injury, while PL infiltration has reduced to baseline by day 7 in retinas treated with FTY720 show no significant PL infiltration and Cx3cr1^+^ cells are evenly distributed throughout the retinal parenchyma. Inserts of each picture show morphological stages of Cx3cr1^+^ cell activation in untreated and FTY720-treated mice. The arrow indicates the direction in which the damaged site is located. **b**, **c** Quantification of the number of GFP^+^ cells and RFP^+^ PL per lesion of untreated and FTY720-treated Cx3cr1^GFP^Ccr2^RFP^ retinas before injury (baseline) and at pre-defined time points (days 0, 1, 4, 7, 10 and 14). Significant differences (**p < 0.01 and ****p < 0.0001) between untreated and FTY720-treated mice were determined by using a post hoc Bonferroni one-way ANOVA test (*n* = 8). **d** Quantification of the polarization coefficient of untreated and FTY720-treated resident immune cells after injury (day 0) and at pre-defined time points (days 1, 4, 7, 10 and 14). Significant differences (****p < 0.0001) between untreated and FTY720-treated mice were determined by using a post hoc Bonferroni one-way ANOVA test (*n* = 8). For both groups, day 0 was chosen as calibrator [NRQ (normalized relative quantification) = 1]. **e** Quantification of the primary and terminal processes per cell after injury (day 0) and at pre-defined time points (days 1, 4, 7, 10 and 14). Significant differences (*p < 0.05) between untreated and FTY720-treated mice were determined by using a post hoc Bonferroni one-way ANOVA test (*n* = 8). Field of view is ≈425 µm

We evaluated in vivo the dynamic response of resident immune cells and PL to laser-induced injury in Cx3cr1^GFP^Ccr2^RFP^ mice, which express RFP in PL, and GFP in resident immune cells including microglia and perivascular macrophages [19, 21, 22, 24, 38–41]. We found persistent inflammation upon injury (Fig. 1). Starting from day 1 after laser coagulation, GFP^+^ and RFP^+^ cells clustered within the lesion and the density of both GFP^+^ and RFP^+^ cells remained significantly elevated for 7 days after injury (Fig. 1a–c). To test if signaling between resident and blood-borne immune cells is involved, we lowered the number of circulating immune cells by FTY720 administration (Additional file 4: Fig. S4a–d). Daily FTY720 treatment caused a reduction of PL in the circulation, including T cells (by ~ 56%) and macrophages, monocytes and neutrophils (by ~ 33%; Additional file 4: Fig. S4a–c). Furthermore, PL clustering in the injury was greatly reduced following drug administration, suggesting that blocking S1p1 through FTY720 treatment interfered with the immune cell recruitment to the inflammatory sites (Additional file 4: Fig. S4d). PL reduction results in earlier resolution of the inflammatory response, as evidenced by GFP^+^ and RFP^+^ cell accumulation in the lesion returning to baseline levels by day 7 (Fig. 1a–d).

In response to insult, Cx3cr1^+^ cells display a remarkable degree of phenotypic plasticity in terms of morphology and branching orientation, which reflects their activation status [42]. To quantify the morphological changes of Cx3cr1^+^ cells and describe the direction of their processes with respect to the location of the focal injury, we determined their polarization coefficient and the number of processes per cell [37]. Before injury, Cx3cr1^+^ cells were in a quiescent state, characterized by ramified processes with no preferential direction and relatively small cell bodies, in both untreated and FTY720-treated mice. During injury response, Cx3cr1^+^ cells became active and underwent morphological changes toward an activated phenotype (Fig. 1a). Untreated mice showed a lower number of processes characterized by increased lengthening of processes oriented towards the injured site at both day 1 and day 7, indicating pro-inflammatory status [43] (Fig. 1a, d).

In contrast, by day 7, Cx3cr1^+^ cells assumed a rounded macrophage-like morphology with enlarged cell bodies and presented more processes in FTY720-treated mice than in untreated animals (Fig. 1a, d). This morphological transformation is associated with their phagocytic activity [44].

In vitro and ex vivo studies showed that FTY720 can modulate cell polarization [45]. Thus, we tested if the relationship between cell polarization and inflammatory responses persists in vivo through correlation analysis (Additional file 5: Fig. S5, Additional file 6: Fig. S6). The polarization coefficient of untreated Cx3cr1^+^ cells was associated only with their clustering in the injured site (Additional file 5: Fig. S5). In contrast, cell polarization in FTY720-treated mice showed a negative correlation with Cx3cr1^+^ cells clustering and a positive one with PL recruitment in the injury, which was reduced by the pharmacological treatment (Additional file 6: Fig. S6). The combination of in vivo imaging of the immune response and its statistical interpretation suggested that FTY720 reduced PL and affected the persistent and harmful inflammatory response to laser-induced injury. Furthermore, Cx3cr1^+^ cells underwent a morphological transformation associated with their phagocytic activity only after FTY720 treatment, which impacted PL response to injury.

**Fig. 2:**
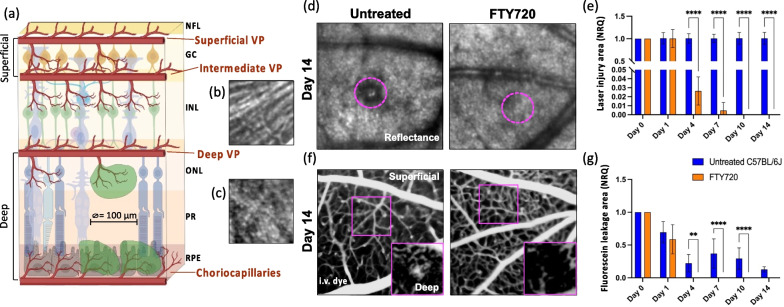
Protective effect of FTY720 treatment on retinal parenchyma and vascular network (**a**–**c**). **a** Schematic of neuronal and vascular remodeling upon laser-induced injury. The injury is represented by a gap in the RPE and PR, where fluorescein leaks from the choroidal microvasculature. We evaluated the BRB breakdown, discriminating between superficial vasculature by the NFL (superficial and intermediate vascular plexus) and the deep vasculature by PR and RPE (deep vascular plexus and choriocapillaris). **b** NFL was identified by the striped-pattern in reflectance (back-scattered light). **c** PR were identified as an increased brightness while focusing into the deeper retina and appearance of the PR mosaic. *NFL* nerve fiber layer, *VP* vascular plexuses, *GC* ganglion cell layer, *INL* inner nuclear layer, *ONL* outer nuclear layer, *PR* photoreceptor layer, *RPE* retinal pigment epithelium. **d**, **e** Kinetics of retinal injury in reflectance in untreated and FTY720-treated mice. The damage was identified as a hypo-reflective circle (≥ 10% lower intensity compared to healthy parenchyma) surrounding a hyper-reflective core (≥ 10% higher intensity compared to healthy parenchyma). **d** Images show the absence of hypo- or hyper-reflectivity in FTY720-treated retinas on day 14, unlike untreated retinas in which the injury was visible (delimited by magenta dashes). **e** Quantification of the damaged area of untreated and FTY720-treated retinas after injury (day 0) and on days 1, 4, 7, 10 and 14. Significant differences (****p < 0.0001) between untreated and FTY720-treated mice were determined using a post hoc Bonferroni one-way ANOVA test (*n* = 8). **f**, **g** Angiographs of untreated and FTY720-treated eyes on day 14. Deep leakage was observed only in untreated animals’ angiograms. **g** Quantification of leakage deep in the retina after injury (day 0) and on days 1, 4, 7, 10 and 14. Significant differences (**p < 0.01 and ****p < 0.0001) between untreated and FTY720-treated mice were determined using a post hoc Bonferroni one-way ANOVA test (*n* = 8). Day 0 was chosen as the calibrator [NRQ (normalized relative quantification) = 1]. Field of view is ≈425 µm

To examine how PL affected retinal repair, we compared the extent of the injury to the retinal parenchyma and the vascular supply upon laser-injury between untreated and FTY720-treated mice (Fig. 2). Within the first 24 h, we detected the edge of the damaged site as a hyper-reflective core surrounded by a hypo-reflective zone similarly in both groups (Fig. 2a, d, e). Whereas the injury was visible throughout the duration of the experiment in untreated retinas, FTY720-treated mice showed a significant reduction of the damage extent starting from day 4 (Fig. 2d). No hypo- or hyper-reflectivity was found in the FTY720-treated retinas on days 10 and 14 (Fig. 2d, e).

In parallel, we investigated the integrity of the vascular network during injury response by adapting FA, an examination performed routinely in the ophthalmologist’s office, to our imaging system. We recorded in vivo the dynamics of fluorescein leakage immediately after injection into the right retro-orbital plexus while the SLO imaged the left retina. We then assessed the breakdown of the BRB, discriminating between superficial vasculature in the proximity of the nerve fiber layer (superficial and intermediate vascular plexus) and the deep vascular network in the proximity of the photoreceptor layer and retinal epithelium (RPE; deep vascular plexus and choriocapillaris; Fig. 2a, f, g).

Laser application resulted in a comparable accumulation of sub-retinal leakage between untreated and FTY720-treated mice 24 h after injury (Fig. 2f, g). The vascular network adjacent to the RPE was compromised until the last time point investigated in the untreated mice, while the superficial vasculature remained intact (Fig. 2f). However, no dye leakage was observed in fluorescein angiograms of FTY720-treated animals starting from day 4 (Fig. 2g). In vivo imaging results were supported by Spearman’s correlations of injury area or dye leak with Cx3Cr1^+^ and Ccr2^+^ cells in untreated mice. The clustering of PL in the lesion is associated with persistent retinal and vascular damage (Additional file 7: Fig. S7).

Overall, these data suggest that FTY720 can contribute to restoring the integrity of the retinal parenchyma and microvasculature by reducing PL infiltration that in turn is associated with the morphological transformation of resident immune cells to amoeboid.

### Adaptive immune response to laser-induced injury in mouse and human retina during degeneration

**Fig. 3:**
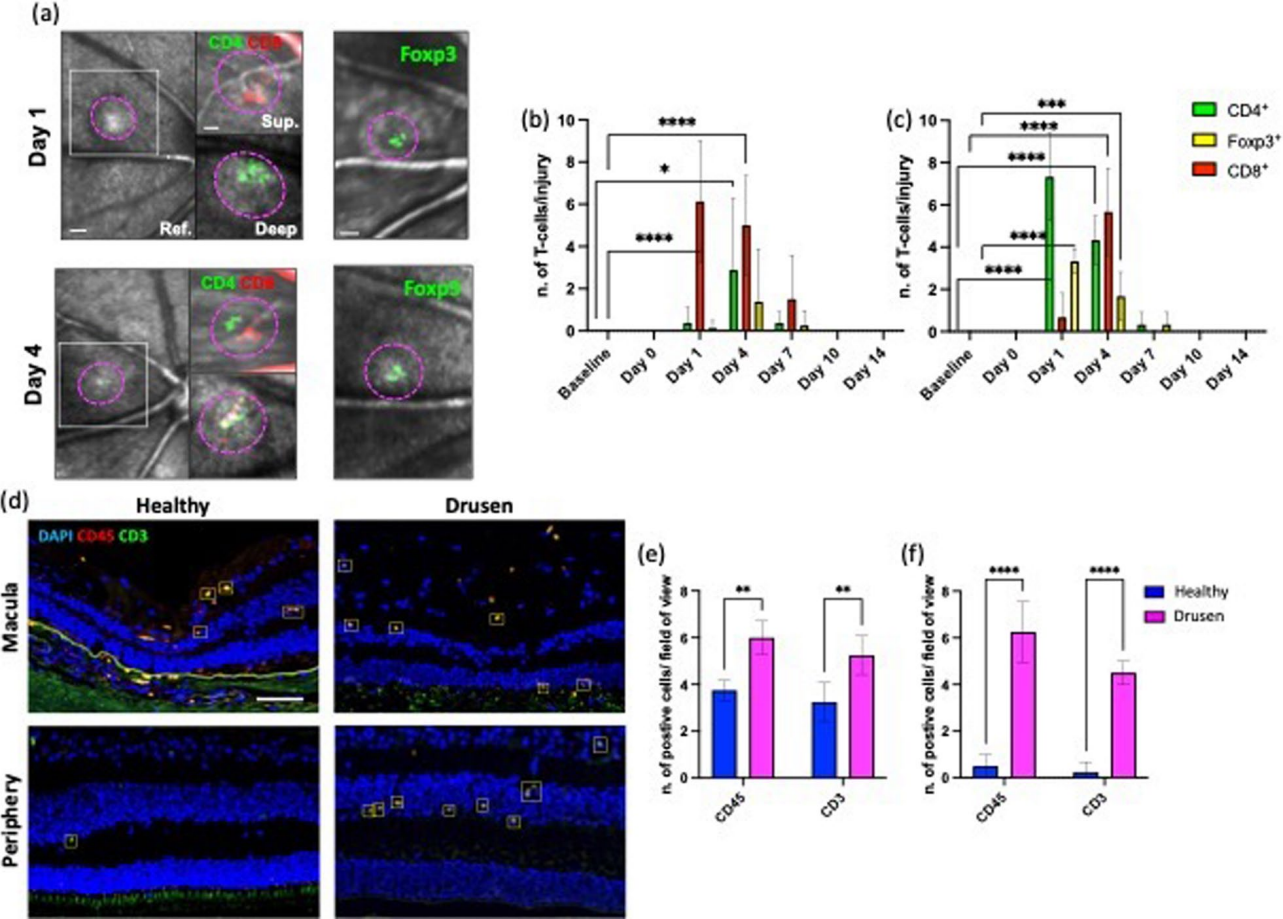
T-cell response to laser-induced injury in mice and during degeneration in human retina. **a**–**c** Tracking of CD4^+^ and CD8^+^ T cells clustering in the same eye of a C57BL/6 mouse and imaging of Foxp3^+^ cells in the same eye of a Foxp3 GFP knock-in mice. **a** Magenta dashes outline retinal injury in reflectance (Ref.), while inserts correspond to the area delimited by a white box in reflectance. Inserts show T cells by the damaged area on days 1 and 4 in the proximity of the NLF (Sup.) and the PR (Deep). Scale bar is 100 μm in reflectance images, 50 μm in the inserts of CD4 and CD8 images and 100 μm images depicting Foxp3 cells. **b** Quantification of the number of CD4, CD8 and Foxp3 T cells per lesion by the superficial vasculature at baseline and at different time points after laser (Da 1, 4, 7, 10 and 14). Significant differences (***p < 0.001 and ****p < 0.0001) between baseline and the different time points were determined by using a post hoc Bonferroni one-way ANOVA test (*n* = 8) **c** Quantification of the number of CD4, CD8 and Foxp3 T cells per lesion found by the deep vasculature network at baseline and on days 1, 4, 7, 10 and 14. Significant differences (***p < 0.001 and ****p < 0.0001) between baseline and different time points were determined using a post hoc Bonferroni one-way ANOVA test (*n* = 8). **d**–**f** Analysis of the inflammatory response in retinas presenting drusen (Drusen) compared to healthy retinas (Healthy) using a pan-PL marker (CD45) and a T-cell co-receptor marker (CD3). **d** Shown are representative sections stained for CD45 (red) and CD3 (green). The number of CD45^+^/CD3^+^ cells is significantly higher in the macula and peripheral retina with drusen. **e** Mean ± SD of the number of CD45^+^ and CD3^+^ cells in the macula. **f** Mean ± SD of the number of CD45^+^ and CD3^+^ cells in the peripheral retina. Significant differences (**p < 0.01 and ****p < 0.0001) between “Healthy” and “Drusen” groups were determined by using a post hoc Bonferroni one-way ANOVA test (*n* = 8). Scale bars equals 100 μm

Although innate immunity and its impact on retinal degenerations are widely explored, the delineation of adaptive immune milieu among the broad leukocyte infiltrate is poorly described in the eye. However, we have visual evidence of the absence of T cells in uninjured tissue and the presence of T cells in the retina 4 days after injury (Additional file 8: Fig. S8). Thus, we dissected the PL into their main lymphoid subpopulation, helper (CD4^+^), regulatory (Foxp3^+^) and cytotoxic (CD8^+^) T cells, and analyzed in vivo their recruitment in the retina upon laser-injury (Fig. 3a–c). We visualized CD4^+^ and CD8^+^ T cells by retro-orbital injection of 5 µg of fluorescently labeled antibodies (AF488 anti-mouse CD4 and AF647 anti-mouse CD8). We detected regulatory T cells using Foxp3^GFP^ mice, which express GFP in regulatory T cells (Fig. 3a–c). T cells were recruited close to the damaged area on days 1 and 4, both in the inner retina near the nerve fiber layer and in the outer retina adjacent to the RPE. CD8^+^ cells clustered on days 1 and 4 above the damage in the proximity of the nerve layer, while helper T cells migrated in the proximity of the injured photoreceptors on day 4. We also observed a nonsignificant number of Foxp3^GFP^ cells on day 4 near the nerve fiber layer close to the injury site (Fig. 3a, b). On the other hand, we found CD4^+^ and Foxp3^GFP^ cells on days 1 and 4 by the injured photoreceptor layer and RPE, followed by the accumulation of CD8^+^ T cells mostly on day 4 (Fig. 3a, c). Interestingly, both CD4^+^ and CD8^+^ cells clustered in the injured area on day 4, concurrent with the earliest differences in the inflammatory response and tissue repair between untreated and FTY720-treated mice (days 7–14; Figs. 1, 2).

We investigated if the lymphocyte recruitment observed in mice also happened during retinal degeneration in humans (Fig. 3d–f). As previously published [29], we sectioned retinal post-mortem samples from 70 to 90-year-old human donors either with healthy cuboidal RPE (Healthy) and retinas or with drusen (Drusen), an early hallmark of age-related macular degeneration (AMD), a retinal degenerative disease [46]. Both groups were subjected to testing for a pan-PL marker known as CD45, as well as a T-cell co-receptor called CD3. In the retinas of elderly patients from both groups, PL was observed, predominantly in the macula, consisting of mostly T cells (~ 97%). Among healthy retinal specimens from elderly patients, 75% exhibited at least one T cell in the macula, while only 25% showed T cells in the periphery. However, the number of CD45^+^/CD3^+^ cells was significantly higher in the macula with drusen when compared to healthy retinas. It was found that every retina displaying drusen also exhibited an increased number of PL, with T cells making up the majority (~ 67%, as shown in Fig. 3d, f). These findings collectively suggest the involvement of adaptive immunity in retinal degenerative diseases in humans, similar to the response seen in laser-induced injury in mice. We have taken advantage of recent advances.

**Fig. 4:**
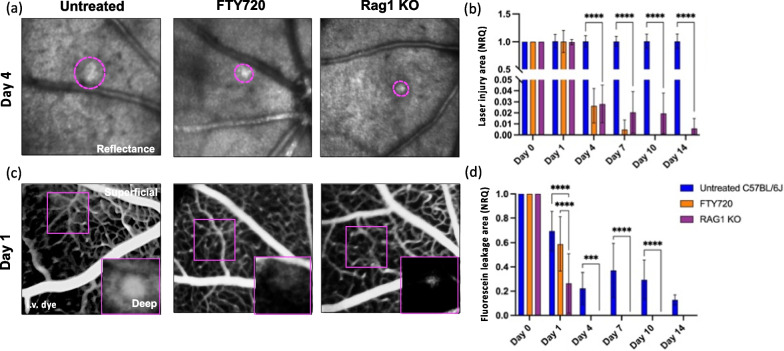
Genetic depletion of lymphocytes accelerated vascular recovery compared to broad PL reduction. **a**, **b** Kinetics of retinal injury detected in reflectance in untreated, FTY720-treated and Rag1 KO mice. Untreated and FTY720-treated mice are the same cohorts shown in Fig. 2. **a** Images show a reduction in the size of hypo- and hyper-reflectivity in the damaged site of FTY720-treated and Rag1 KO retinas on day 4 unlike untreated retinas. **b** Quantification of the damaged area of untreated and FTY720-treated, and Rag1 KO retinas after injury (day 0) and at pre-defined time points (days 1, 4, 7, 10 and 14). Significant differences (**p < 0.01 and ****p < 0.0001) between untreated and FTY720-treated mice were determined by using a post hoc Bonferroni two-way ANOVA test (*n* = 8). **c** Representative fluorescein angiographs of untreated and FTY720-treated, and Rag1 KO eyes on day 1. **d** Quantification of dye that leaks deep in the retina, identified as leakage area, after injury (day 0) and at pre-defined time points (days 1, 4, 7, 10 and 14). Significant differences (**p < 0.01 and ****p < 0.0001) between untreated, FTY720-treated and Rag1 KO mice were determined by using a post hoc Bonferroni two-way ANOVA test (*n* = 8). For both groups, day 0 was chosen as calibrator [NRQ (normalized relative quantification) = 1]. Field of view is ≈425 µm

So far, we showed that lymphocytes infiltrate the retina in mice during injury response. Next, we address the question if the sped-up recovery observed in FTY720-treated mice arises from T-cell depletion. To this end, we employed Rag1 KO mice in which lymphocytes are absent in the whole body. We compared the area of the injured parenchyma and the vascular leak upon laser-injury among untreated, FTY720-treated and Rag1 KO mice (Fig. 4). Similar to FTY720-treated mice, Rag1 KO mice showed a reduction in the size of the damaged area compared to untreated mice (days 4–14; Fig. 4a, b), indicating that absence of lymphocytes has the same beneficial effect on recovery of retinal parenchyma from injury as a broad PL reduction. A minimal area of hypo- and hyper-reflectivity was found in Rag1 KO retinas on days 10 and 14, which was not statistically significantly different from that found in FTY720-treated retinas (Fig. 4b). Furthermore, genetic depletion of lymphocytes accelerated recovery of the vascular network compared to FTY720-treated mice. Minimal fluorescein leakage was visible in Rag1 KO mice (Fig. 4c, d). These data suggested that genetic depletion of lymphocytes improved retinal repair even compared to the broad PL reduction.

### Impact of CD4^+^ and CD8^+^ T-cell infiltration on inflammatory response and tissue repair after laser-induced injury

**Fig. 5:**
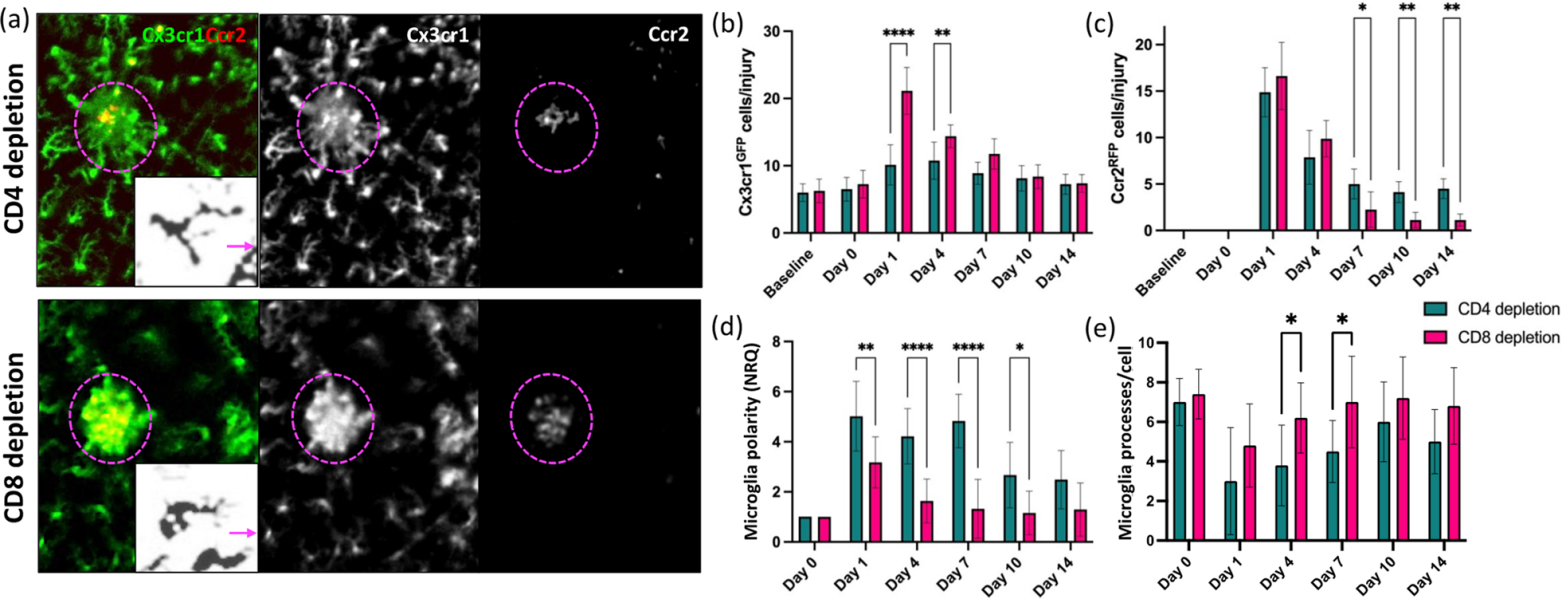
Beneficial effect of CD8 depletion on resident immune cell activation and PL recruitment. **a**–**d** Inflammatory response of CD4- and CD8-treated Cx3cr1^GFP^Ccr2^RFP^ mice. **a** Images show differences resident and blood-borne immune cell recruitment on day 1 in the damaged site (delimited by magenta dashes). Anti-CD4 treatment lessened Cx3cr1^+^ cell recruitment to the damaged area. Inserts show morphological stages of Cx3cr1^+^ cell activation in anti-CD4- and CD8-treated mice. The arrow indicates the direction in which the damaged site is located. **b**, **c** Quantification of the number of GFP^+^ cells and RFP^+^ PL per lesion of anti-CD4- and CD8-treated Cx3cr1^GFP^Ccr2^RFP^ retinas before injury (baseline) and at pre-defined time points (days 0, 1, 4, 7, 10 and 14). Significant differences (*p < 0.1, **p < 0.01 and ****p < 0.0001) between anti-CD4- and CD8-treated mice were determined by using a post hoc Bonferroni two-way ANOVA test (*n* = 8). **d** Quantification of the polarization coefficient of anti-CD4- and CD8-treated resident immune cells after injury (day 0) and at pre-defined time points (days 1, 4, 7, 10 and 14). Significant differences (*p < 0.1, **p < 0.01 and ****p < 0.0001) between anti-CD4- and CD8-treated mice were determined by using a post hoc Bonferroni one-way ANOVA test (*n* = 8). For both groups, day 0 was chosen as calibrator [NRQ (normalized relative quantification) = 1]. **e** Quantification of the primary and terminal processes per cell after injury (day 0) and at pre-defined time points (days 1, 4, 7, 10 and 14). Significant differences (*p < 0. 1) between anti-CD4- and CD8-treated mice were determined by using a post hoc Bonferroni one-way ANOVA test (*n* = 8). Field of view is ≈425 µm

To further specify the T-cell subpopulation affecting retinal injury response, we analyzed how CD4^+^ and CD8^+^ T cells affect the time course of resident and blood-borne immune cell response to injury in the retina (Fig. 5). To deplete either CD4^+^ or CD8^+^ T cells selectively, we injected specific monoclonal antibodies (CD4 GK1.5 or CD8 2.43) for 3 consecutive days before injury and on day 8 following injury. Anti-CD4 treatment significantly reduced Cx3cr1^+^ cell recruitment in the damaged area, while the density of GFP^+^ cells was comparable to untreated mice between days 1 and 14 in anti-CD8-treated mice (Fig. 5a, b). In both groups, PL migrated toward the lesion starting from day 1, and the number of RFP^+^ cells in the injury was comparable for 4 days (Fig. 5a, c). However, PL density after anti-CD4 treatment was higher than in the anti-CD8-treated group from day 7 to 14 (Fig. 5c).

We next quantified the morphological changes of GFP^+^ cells and compared their polarization toward the injury between CD4- and CD8-treated mice (Fig. 5a, d). Before injury, Cx3cr1^+^ cells were similar in a resting state in both groups, with small cell bodies and ramified processes with no preferential direction. Cx3cr1^+^ cells in CD4-treated mice were characterized by increased lengthening of fewer processes oriented towards the injured site, indicative of a pro-inflammatory status (Fig. 5a, d). In contrast, Cx3cr1^+^ cells in CD8-treated mice assume a rounded macrophage-like morphology with more processes than in CD4-treated animals, and this morphological transformation has been associated with phagocytic activity [44] (Fig. 5a, d).

**Fig. 6:**
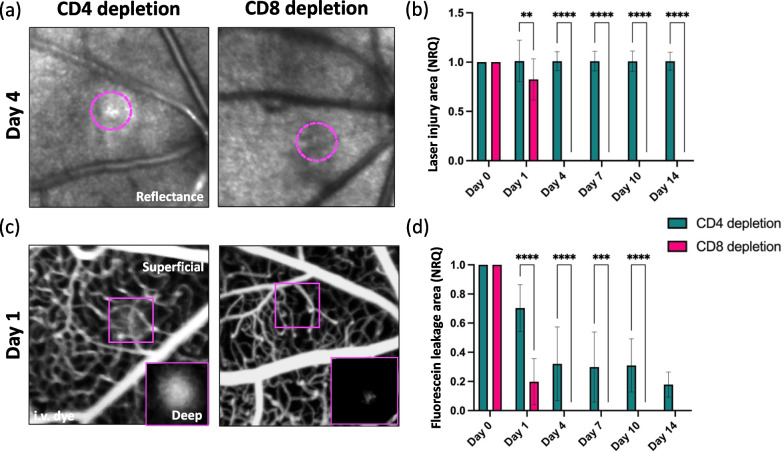
Protective effect of CD8 depletion on lesion recovery and BRB regeneration. **a**, **b** Kinetics of retinal injury detected in reflectance in CD8- and CD4-depleted mice. **a** Images show the absence of hypo- and hyper-reflectivity in the damaged site of CD8-depleted retinas on day 4, whereas the injury was visible in CD4-depleted retinas (delimited by magenta dashes). **b** Quantification of the damaged area of anti-CD8- and CD4-treated retinas after injury (day 0) and at pre-defined time points (days 1, 4, 7, 10 and 14). Significant differences (****p < 0.0001) between two groups were determined by using a post hoc Bonferroni one-way ANOVA test (*n* = 8). **c** Representative fluorescein angiographs of eyes treated with anti-CD8- or CD4-antibody on day 1. The leakage area was observed only in fluorescein angiograms of anti-CD4-treated animals, while minimal vascular damage was detected in the depth of CD8-depleted retinas. **d** Quantification of fluorescein that deep in the retina, identified as leakage area, after injury (day 0) and at pre-defined time points (days 1, 4, 7, 10 and 14). Significant differences (**p < 0.01 and ****p < 0.0001) between anti-CD8- and CD4-treated mice were determined by using a post hoc Bonferroni one-way ANOVA test (*n* = 8). For both groups, day 0 was chosen as the calibrator [NRQ (normalized relative quantification) = 1]. Field of view is ≈425 µm

As we did in FTY720 treated and in Rag1 KO mice, we further investigated the role of CD4^+^ and CD8^+^ T cells during retinal repair by comparing the extent of the injured area of the retinal parenchyma and the fluorescein leakage upon laser-injury between groups (Fig. 6). In CD4-depleted mice, we detected a hyper-reflective signal surrounded by a hypo-reflective circle, which outlines the damaged site throughout the experiment. The lesion size remained unchanged (Fig. 6a, b), comparable to untreated mice. The injury was also visible in anti-CD8-treated mice on day 1, but a lesion was consistently no longer detected starting from day 4 (Fig. 6a, b). In vivo FA confirmed CD4 depletion might be detrimental to injury response in the retina, and accumulation of sub-retinal fluid was found throughout the experimental period of 14 days **(**Fig. 6d). On the other hand, CD8 depletion significantly reduced fluorescein leakage already 24 h after injury. Fluorescein leakage was absent in CD8-depleted animals starting from day 4 (Fig. 6c, d).

In vivo data were supported by Spearman's correlations of injury or leak area with CD4^+^ and CD8^+^ T cells in untreated mice (Additional file 9: Fig. S9). We showed an association between CD8^+^ T-cell clustering in the injury and retinal and vascular damage, especially on day 4, when CD8^+^ T cells peak in the injury. CD4^+^ T cells do not associate with the extent of either injured parenchyma or fluorescein leakage. These data suggest a harmful effect on retinal repair specifically by CD8^+^ T cells.

Together these data suggest that CD4 is necessary for regulating the proper immune response to retinal damage. Its depletion reduced resident immune cell migration to the injury site and favored their pro-inflammatory phenotype, which may trigger a prolonged PL infiltration. CD4 depletion prolonged the existence of the retinal lesion and prevented the recovery of the BRB. Conversely, CD8 depletion may be beneficial for the immune response to retinal damage. CD8 depletion resulted in Cx3cr1^+^ cells assuming a phagocytic phenotype and relative reduction in PL infiltration. Similarly, both laser lesions and BRB recovered much more quickly when CD8 was depleted.

**Fig. 7:**
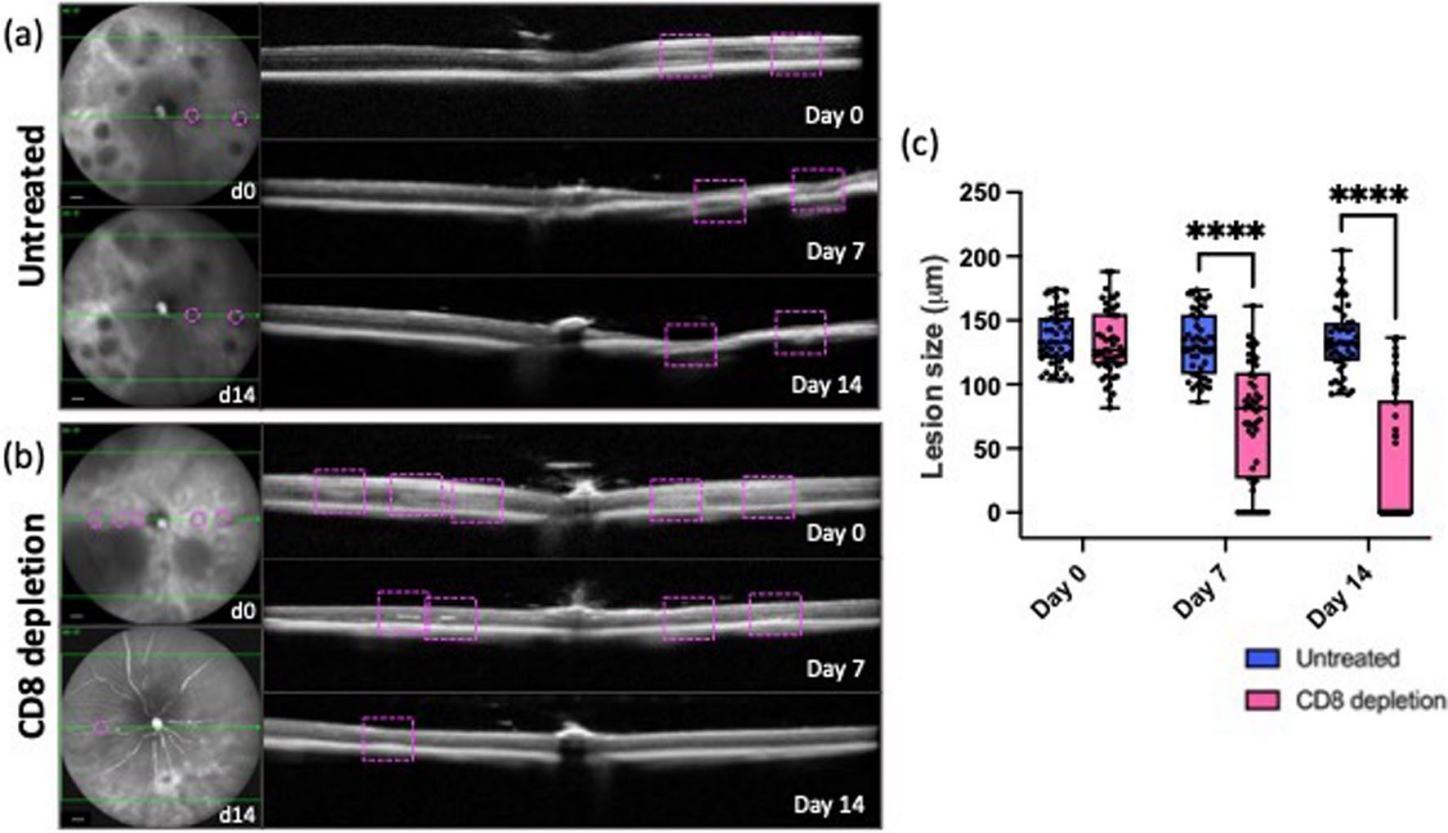
CD8-antibody treatment stimulates tissue repair of the retinal parenchyma. **a**, **b** Near-infrared OCT images (left) and cross-sectional B-scans (right) of injured eyes of the same untreated or anti-CD8-treated animal after injury (Day 0) and on days 7 and 14 after injury. Cross-sectional images were taken at the level of the ONH. Representative lesions are outlined by magenta dashes in the enface view and a magenta dashed box in the cross-sectional scan. **c** Quantification of the width of the laser lesions in the retina cross-section (mean ± SD). Significant differences (****p < 0.001) between untreated and anti-CD8-treated groups were determined by using a post hoc Bonferroni one-way ANOVA test (*n* = 50). Representative scans were selected (bold green line)

To simulate a widespread retina degeneration, we placed multiple laser-injuries into most of the retinal tissue using the Visulas 532 s diode laser. We acquired cross-sectional images of the retina longitudinally by SD-OCT and checked the structure of the retinal layers in untreated compared to anti-CD8-treated mice (Fig. 7). After damaging the retina (day 0), we found diffuse hyper-reflective signals that extended from the RPE to the OPL (outer plexiform layer) similarly in both groups (Fig. 7a–c). Starting from day 7, cross-sections of untreated retinas showed discontinuities in the RPE and OPL layers, and the retina thinned where hyper-reflective signals were previously detected (Fig. 7a). The retinal thickness was further reduced where the tissue was damaged, and the retina's layering was hard to identify in the untreated mice (Fig. 7a). Contrariwise, the hyper-reflective signals were more compact and restricted mainly to the ONL (outer nuclear layer) in the anti-CD8-treated retinas starting from day 7 (Fig. 7b). Interestingly, most of the detectable laser spots were identified as mild bumps due to the thickening of the RPE at the last timepoint analyzed (Fig. 7b). Therefore, we compared the average width of the injuries detected in the cross-sections of untreated and anti-CD8-treated retinas and found a significant reduction of the damaged area starting from day 7. Indeed, the average extent of the injuries halved on day 7 (from ~ 133 to ~ 72 μm) and then reduced to ~ 35 μm at the last timepoint analyzed (day 14, Fig. 7c).

**Fig. 8:**
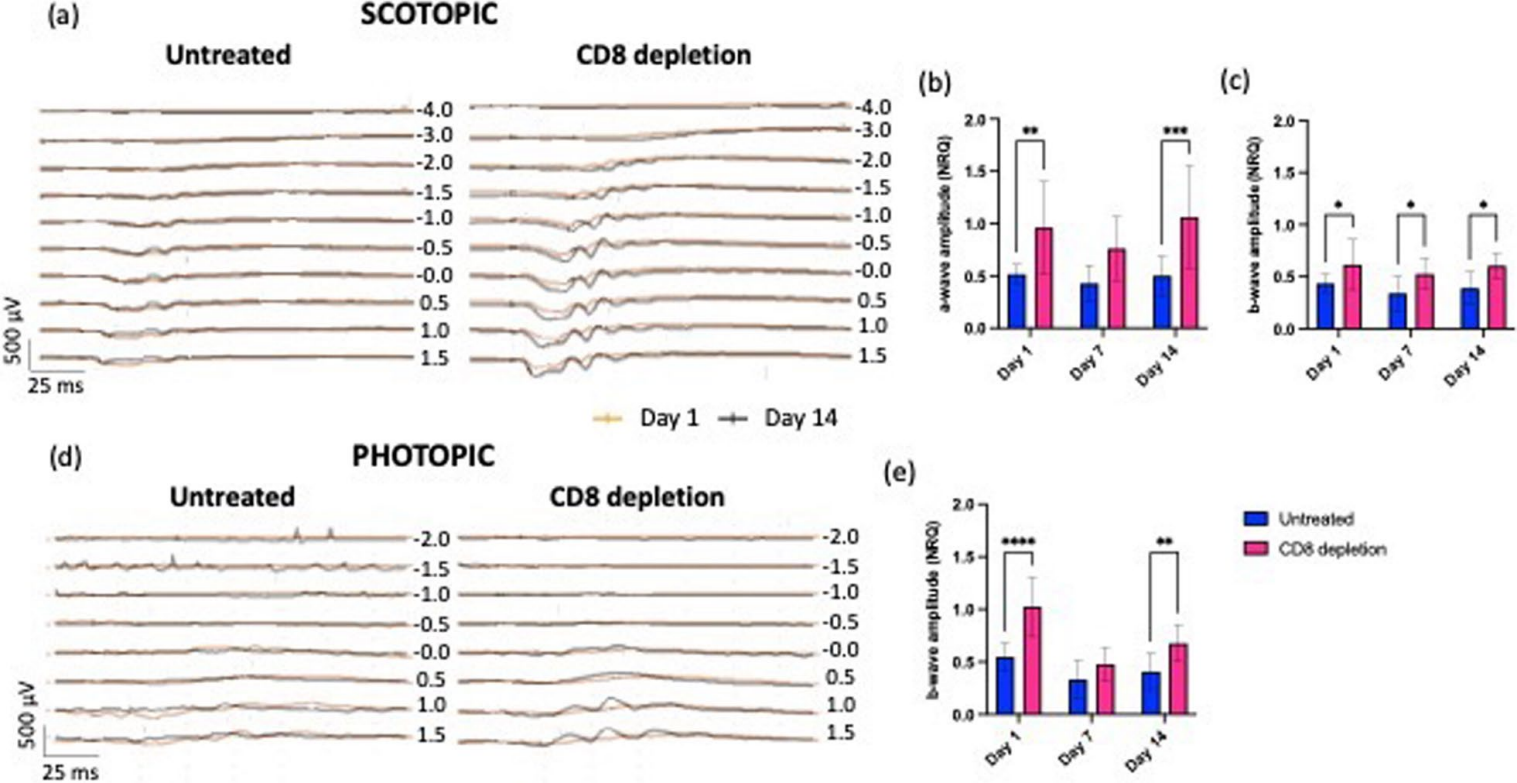
Improved retinal function in mice after anti-CD8 treatment. **a**–**e** Representative scotopic and photopic ERGs recorded with increasing light intensities from dark-adapted untreated and anti-CD8-treated C57BL/6 mice. **a** Scotopic traces on day 14 (black) superimposed on electroretinograms of day 1 of untreated and anti-CD8-treated retinas. **b**, **c** Shown are mean ± SD of scotopic a- (**b**) and b-wave (**c**) amplitudes. **d** Photopic traces on day 14 (black) superimposed on electroretinograms of day 1 of untreated and CD8-treated retinas. **e** Shown are mean ± SD (*n* = 4–5) of photopic b-wave (**e**) amplitudes. For both groups, day 0 was used as calibrator [NRQ (normalized relative quantification) = 1]. Significant differences (*p < 0.1, **p < 0.01, ***p < 0.001 and ****p < 0.0001) between untreated and CD8-treated mica were determined by using a *t*-test (*n* = 4–5)

In anti-CD8-treated retinas, functional tissue recovery was confirmed by ERG. Scotopic and photopic a-/b-wave amplitudes were significantly increased in anti-CD8-treated mice starting already 24 h post-injury (Fig. 8a–e), further supporting a deleterious role for CD8^+^ T cells during injury response in the retina. Although the difference in the a-/b-waves between untreated and anti-CD8-treated groups was not significant on day 7 (Fig. 8b, e), the function of the neurosensory retina improved 14 days after injury only following anti-CD8 treatment unlike in untreated animals (Fig. 8b, c, e).

**Fig. 9:**
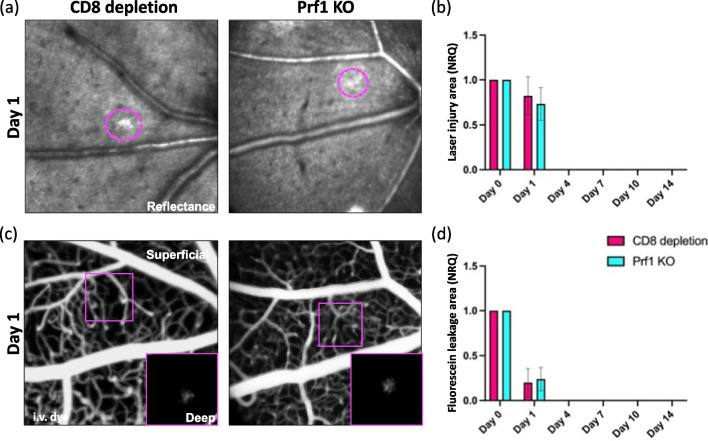
Perforin deletion has the same beneficial effect as the depletion of CD8^+^ T cells. **a**, **b**) Kinetics of retinal injury detected in reflectance in CD8-treated and Prf1 KO mice. Anti-CD8-treated animals were part of the same cohorts in Fig. 6. **a** Images show similar area of hypo- and hyper-reflectivity in the damaged site of Prf1 KO and CD8-treated mice on day 1 (delimited by magenta dashes). **b** Quantification of the damaged area of CD8-treated and Prf1 KO retinas after injury (day 0) and at pre-defined time points (day 1, 4, 7, 10 and 14) (*n* = 8). **c** Representative fluorescein angiographs of CD8-treated and Prf1 KO eyes on day 1. A small accumulation was observed in fluorescein angiograms of Prf1 KO mice, as observed after anti-CD8-antibody treatment. **d** Quantification of dye that leaks in the depth of the retina, identified as leakage area, after injury (day 0) and at pre-defined time points (day 1, 4, 7, 10 and 14) (*n* = 8). For both groups, day 0 was chosen as calibrator [NRQ (normalized relative quantification) = 1]. Field of view is ≈425 µm

CD8^+^ T cells are the primary source of Prf1, a pore-forming cytolytic protein, which induces apoptosis in its target cells. We thus investigated if ablation of Prf1 results in outcomes similar to CD8 depletion using Prf1 KO mice. These mice have normal numbers CD8^+^ T cells that are all Prf1-deficient [47]. We examined the extent of the injured area in the retinal parenchyma and the fluorescein leakage upon laser-injury Prf1 KO mice and compared them to CD8-treated mice (Fig. 9a–d). The injury was similarly detected between Prf1 KO and anti-CD8-treated animals, and the hypo- and hyper-reflectivity was no longer found starting from day 4 (Fig. 9a, b). Furthermore, genetic deletion of Prf1 resulted in a small accumulation of sub-retinal fluid only on day 1, as observed after the depletion of CD8^+^ T cells (Fig. 9c, d). These data suggest that CD8^+^ T cells act via the Prf1 pathway to impair retinal regeneration.

## Discussion

Previous investigations into the immune response to retinal damage have primarily relied on either observing a limited number of cell types at specific time points or sectioning the parenchyma. This has restricted the understanding of dynamic interactions among immune cells with different lineages and functions, as well as the duration and timing of the response phase. However, recent advances in experimental systems have allowed for the real-time investigation of inflammatory dynamics in the retina. By using in vivo microscopy in preclinical mouse models, we can now overcome these limitations and simultaneously characterize the spatio-temporal dynamics of resident and blood-borne immune responses [48–52]. The local innate immune response of resident immune cells after focal tissue challenge has been tracked by in vivo imaging of the brain [53, 54]. However, optical imaging in the brain requires invasive surgical procedures, which can, in itself, generate an injury response that masks the inflammatory mechanisms of interest [55].

Through in vivo retinal imaging, we exploited the eye as a natural window into the CNS to assess non-invasively and longitudinally structural and fluorescence changes at a defined location over hours, days, and weeks in response to a focal laser injury. We have taken advantage of recent advances in mouse retinal imaging to survey resident and blood-borne immune cells in their resting state and during their response to a focal injury. Miller et al. [52, 56] assessed in vivo the response of either Cx3cr1^+^ microglia to laser-induced injury or CCR2^+^ peripheral monocytes to light-induced photoreceptor degeneration. Building upon this knowledge, our in vivo experiments utilizing double-reporter mice to assess the combined inflammatory response of these cell types, and further employed minimally invasive labeling techniques to provide evidence supporting the detrimental role of PL, particularly T cells, in the retina's injury response. Leukopenia, as well as genetic depletion of T cells, reduced injured areas in the parenchyma and improved the BRB integrity. Additionally, Cx3cr1^+^ cells underwent morphological changes toward an amoeboid phenotype during injury response in both conditions. Interestingly, antibody depletion of CD8^+^ T cells, apart from reducing the damaged area like observed with FTY720 treatment and genetic reduction of lymphocytes, accelerated recovery of the BRB compared to broader depletions. After anti-CD8 treatment, the retinal function also improved, concomitant with a beneficial immune response. Collectively, our data from in vivo imaging provide novel insights into the involvement of adaptive immunity in mouse eyes in response to laser-induced injury as well as in humans during retinal degeneration.

The laser-induced injury model has recently shown to be relevant for studying human neurodegeneration in the retina as it mimics certain macroscopic features of human retinal degenerative diseases (e.g., AMD; [29]), such as photoreceptor disorganization and degeneration, proliferation of glial cells and gliotic or fibrotic scarring [52]. Moreover, our injury model is an excellent resource for investigating specific biological processes directly in the area of damage. In support of this, previous studies have provided in vivo evidence that both resident and blood-borne immune cells migrate into the area of injury in the retina following the induction of light-induced retinal lesions. These studies have utilized various techniques, including labeling microglia with fluorescent dyes and using reporter mice for phagocyte lineage cells. With our injury model, researchers can build upon these previous findings and further study the dynamic responses relevant to human retinal degeneration in vivo. Inflammation is considered a critical contributing factor to retinal degenerative diseases; however, complex immune interactions among cells with different lineages, roles, and reactivity to retinal insults are still unclear. Here we provide evidence of the harmful effect of PL during laser-induced injury response in mice. FTY720 treatment has rescued retinal parenchyma and BRB integrity in parallel with an earlier resolution of the inflammatory response. In line with results obtained with our injury model, FTY720 has already shown a protective effect in several diseases because of its impact on immune cell migration and vascular permeability via the S1p1 signaling, e.g., in diabetic retinopathy [57], retinal dystrophy [58], light-induced degeneration [59], and glaucoma [60], choroidal neovascularization [61], uveitis [62], and retinitis pigmentosa [63]. Even if a direct action on vascular endothelial cells cannot be entirely excluded [61], targeting PL offers a promising approach for recovering the retina following damage.

Nevertheless, it is challenging to address if the beneficial impact of FTY720 on retinal repair is due to its impact on PL recruitment or a direct effect on resident immune cell activation. In vitro and ex vivo studies investigated whether FTY720 directly regulated microglial polarization without considering FTY720’s impact on PL recruitment. They have shown immunosuppressive activities in microglia via modulation of the S1p1 [64, 65]. Furthermore, the anti-inflammatory effect of FTY720 was associated with the inhibition of microglial autophagy [66] and the promotion of their phagocytosis [65]. Similarly, for the first time to our knowledge, we showed in vivo that FTY720-treated Cx3cr1^+^ cells react to injury induction, assuming a rounded macrophage-like morphology with enlarged cell bodies and few processes indicative of phagocytic activity [44]. Additionally, their morphological transformation positively correlated only with the reduced PL recruitment in the injured area after the pharmacological treatment. These data imply that PL can act on resident immune cell response to laser-induced injury. However, Spearman's correlation explains relationships between quantitative variables and how strong that relationship may be, not the causality between them. To confirm if PL reduction induced by FTY720 treatment has a direct effect on retinal repair, it would be necessary to use an animal model with non-reactive resident immune cells or a model that lacks them entirely within the retina. However, previous research has shown that even in the presence or absence of PL infiltration, retinas without resident immune cells cannot regenerate [67–69]. Overall, these data suggest that FTY720 contributes to restoring the integrity of retinal parenchyma and microvasculature by acting on PL infiltration that is associated with the morphological transformation of Cx3cr1^+^ cells from resting to amoeboid.

Different clinical trials have been conducted to target the inflammatory pathways in the retina [70], but they have not yet been successful and why some have failed is uncertain. A significant limitation is the relative lack of understanding of the specific role of the different immune cell types within the retinal inflammatory orchestra that leads to tissue degeneration. Genetic studies [28, 71], human histological data [72, 73], and animal studies [74] have primarily focused on the role of innate immunity in mediating cell death after tissue damage in the progression of retinal degeneration. However, it is known that not only the innate immune system but also the adaptive immune system is involved in the pathogenesis of retinal degeneration [10, 75]. Concerning functions of adaptive immunity within the immune response, both CD4^+^ and CD8^+^ T cells act as promoters and regulators of inflammation in the retinal tissue. Indeed, T cells can amplify the immune response by releasing cytokines and chemokines, promoting crosstalk among immune cells accordingly. However, the role of T cells is controversial as specific subpopulations of T cells can also assume a regulatory phenotype and modulate the intensity of the immune response, which is crucial for the resolution phase of the inflammatory process [8, 10]. In our injury model in mice in combination with in vivo imaging, we found cytotoxic T cells in the proximity of the nerve fiber layer on day 1 and only a few helper and regulatory T cells were found on day 4. In addition, we observed in vivo the recruitment of helper and regulatory T cells by the injured photoreceptor layer and RPE on day 1, followed by the further accumulation of cytotoxic cells in the retinal parenchyma on day 4. Within this timeframe, we also found the first difference between the untreated and FTY-treated group in the capacity of the retinal tissue and BRB to repair themselves. Indeed, the damaged area started to shrink significantly from day 4. These data from in vivo imaging provided evidence that both helper and cytotoxic T cells might participate in the immune response to laser-induced injury. It would be important for future studies to assess further to which extent T cells contribute to retinal damage by using different preclinical models of degeneration (e.g., chemical-induced or genetic models) as well as using different ex vivo techniques complementary to our in vivo experiments.

Other studies have shown the involvement of adaptive immunity in the retinal pathology of various disease and injury models in mice [76–78]; however, little is known about the contribution of T cells to retinal degeneration in humans. Most research has been done on peripheral blood or comparing drusen-presenting choroid with non-drusen tissue [13, 79]. For the first time to our knowledge, we analyzed samples of retinal parenchyma from human donors between 70 and 90 years old either with healthy retinas with cuboidal RPE or retinas with drusen. Although both groups presented T cells in the macula, a significantly higher number of T cells resided in the macula with drusen. In the peripheral retina, T cells were rarely found in healthy eyes but were abundant in eyes with drusen. We presume that T cells were present in the healthy macula due to age-related changes, but difficulties in retrieving retinal samples from young donors make it challenging to verify this assumption. Recently, experimental findings pointed to higher transmigration of T cells as part of an amplified response of the aging CNS, and data in rodents indicate a gradual increase of T-cell-homing into the brain in the steady state starting in middle age [80–82]. Altogether, our data indicate that the adaptive immune system participates in the injury response in mice. In human retinas, cells of the adaptive immune system (T cells) appear to accumulate with age and during degeneration.

We then analyzed the impact of T cells on retinal repair using a mouse model lacking mature T cells, Rag1 KO mice [83]. *Rag1* is required to rearrange antigen receptor genes of lymphocytes [84]. However, it is also present in the murine and human CNS in areas of high neural density of the brain (e.g., cerebellum, hippocampus) and the retina. In particular, *Rag1* plays a role in programmed cell death in the visual system, but it was detected solely in retinal ganglion cells in a model resembling features found in human glaucoma [85–88], but not in photoreceptors that are the target of our injury model. Upon injury, Rag1 KO mice showed a reduction of the damage to the retinal parenchyma by day 4, which is similar to what we observed after FTY720 treatment against all PL. Genetic depletion of lymphocytes also accelerated recovery of the BRB compared to leukopenia, and our results are supported by the well-accepted role of blood-borne lymphocytes on CNS vascular barriers (e.g., BRB, blood–brain barrier or BBB). Infiltrating T cells can increase vascular permeability, as they cause microvascular disorder and secrete inflammation-associated molecules [89].

However, T cells are composed of heterogeneous subpopulations. A unique subpopulation of helper T cells, called Foxp3^+^ regulatory T cells or Tregs, diminished the harmful action of other immune cells (e.g., monocytes, macrophages) on CNS barriers by various means, such as by cell-to-cell contact and the release of suppressive cytokines. Thus, Tregs can promote BRB and BBB repair and suppress angiogenesis [90–92].

We then depleted the helper T cells and cytotoxic T cells using antibody treatment against CD4 or CD8, respectively, in order to better understand the role of T cell subpopulations on local inflammation and repair. Depletion of CD4^+^ T cells reduced the resident immune cell accumulation at the injury site and prolonged PL infiltration. Consequently, the retina could not initiate reparative processes: the damaged site and an accumulation of sub-retinal fluid were visible throughout the experiment. These results resemble the injury response observed in untreated mice. On the other hand, blocking CD8^+^ T-cell-mediated immune responses led to the accumulation of phagocytic-like immune cells and an accelerated resolution of PL in the damaged retina by day 7, as well as a faster BRB recovery. No damage was outlined in the retinal parenchyma by day 4, when we detected CD8^+^ cell migration to the injured photoreceptor layer and RPE. Furthermore, fluorescein leakage was minimized by depleting CD8^+^ T cells within 24 h after laser-injury induction. At the same time, we found the recruitment of CD8^+^ T cells in the proximity of the nerve fiber layer. These data indicate that CD4^+^ and CD8^+^ T-cell subsets infiltrate the retina in response to injury, leading to different results.

We observed a pronounced effect on neuroinflammation despite the relatively low number of T cells infiltrating the retina after a focal injury, especially when compared to the much larger cell count of innate immune cells, both invading and resident, in the damaged eye. Nonetheless, similar phenomena have been documented in other contexts, such as in the aftermath of a stroke in the brain [93]. Hence, we suppose that the scale of the impact on inflammation and retinal repair is not directly attributable to the number of cells recruited to the injury site, but rather to their hyper-local influence within a laser lesion of ≈100 µm diameter.

The CD4^+^ subpopulation plays a critical key role in immune regulation, and their pharmacological depletion impacts tissue repair. In particular, resident immune cell response to injury was affected, as fewer Cx3cr1^+^ cells were recruited in the damaged area and showed an increased lengthening of processes oriented towards the injured site, indicative of a pro-inflammatory status. Our results are in line with the literature [94, 95] documenting the interaction between reactive Cx3cr1^+^ cells and CD4^+^ T cells in various neurodegenerative disease models. Interestingly, microglial phenotype results from reciprocal signaling and microglia-T cell interactions. For instance, CD4^+^ T cells can actively contribute to the local immune response by acting directly on the microglia and suppressing their production of reactive oxygen species [96–99]. Conversely, changing microglial phenotype may affect T cell response to neuronal damage [81, 100]. For instance, the pharmacological inhibition of microglial reactivity markedly affected the extent of T cell infiltration [101]. Furthermore, the absence of a CD4^+^ T-cell-mediated immune response promoted PL infiltration on days 7 and 14 in our injury model. This may be due to pro-inflammatory-like microglia upregulating the production of chemokines, which thus favors the recruitment of immune cells to the retinal parenchyma [102]. The overall result is a maladaptive immune response that impeded tissue and BRB recovery.

CD8^+^ T cells also interact with the resident immune cells through reciprocal signaling. Microglia can positively and negatively regulate the recruitment of CD8 T cells in the CNS parenchyma [103, 104]. Reciprocally, infiltrating CD8^+^ T cells are responsible for phenotypic changes in microglia as CD8^+^ cells recruitment in the damaged area is associated with a pro-inflammatory environment in the CNS parenchyma, which alters the activation state of microglia into a pro-inflammatory way [105]. Indeed, we showed that depletion of CD8^+^ T cells modified the immune response locally in the retina by confining the resident and blood-borne immune cell recruitment in the injury to only 4 days. Furthermore, Cx3cr1^+^ cells underwent morphological changes toward a phenotype previously shown to be associated with a more phagocytic and anti-inflammatory state during the early immune response. Altogether, unlike anti-CD4-antibody treatment, depletion of CD8^+^ T-cell-mediated immune responses contributed to a rapid and beneficial immune response resulting in the repair of retinal parenchyma and BRB recovery.

We then determined if antibody treatment against cytotoxic T cells stimulates retinal repair and functional recovery of the retina upon damaging most of the outer retina. As we observed after a single laser burn, CD8-antibody treatment enables restoration of the retinal structure and the re-establishment of the retinal layering compared to the untreated control. Indeed, cross-sectional B-scans of anti-CD8-treated eyes showed a reduction in the number of laser spots identified as mild bumps in the RPE layer on day 14. Conversely, untreated retinas thinned over time in the region of the laser lesions to such a degree that identifying retinal layering was no longer possible. Furthermore, the electrophysiological responses detected with ERG after CD8 T-cell depletion showed accelerated recovery compared to untreated controls. Target rod- and cone-pathway function (scotopic and photopic ERG, respectively) improved starting from 24 h after injury, corresponding to the timing of a lower accumulation of sub-retinal fluid in the angiograms recorded by in vivo imaging after a single laser burn. Statistical interpretation of our imaging results further strengthened our findings in vivo and electrophysiological measurements. Indeed, we found a significantly positive correlation between cytotoxic T cells and retinal and vascular damage, suggesting that the infiltration of CD8^+^ T cells and their subsequent clustering in the damaged area prevents lesion recovery. Altogether, our results show that CD4^+^ and CD8^+^ T cells can affect the immunological equilibrium in a supportive or pathogenic manner once they infiltrate the injured retina, similar to what Daglas et al. [33] showed in the brain after traumatic brain injury (TBI). Indeed, they demonstrated that either genetic deficiency or pharmacological depletion of CD8^+^ T cells, but not depletion of CD4^+^ T cells, ameliorates neurological outcomes, indicating the detrimental role of cytotoxic T cells post-TBI.

Finally, we aimed to understand by which mechanism cytotoxic T cells can influence the remodeling of retinal tissue and BRB. Although CD8^+^ T cells also produce cytokines, such as IFN-γ, they are best known for their cytolytic functions. This T-cell subset contains granules with Prf1, which are delivered to the target cells to induce cell death via membrane damage and cellular content release [106]. Our data are consistent with the idea that CD8^+^ T cells mediate retinal damage via the Prf1 pathway. Genetic deletion of Prf1-mediated immune response in mice with standard numbers of CD8^+^ T cells reproduced similar results to antibody treatment against cytotoxic T cells. Indeed, the injured area was no longer found starting from day 4 in the retinal parenchyma concomitant with a lack of sub-retinal fluid accumulation in fluorescein angiograms of Prf1 KO mice, consistent with the results obtained after depletion of CD8^+^ T cells. These data suggest CD8 T cells act via the Prf1 pathway to impair retinal and BRB repair mechanisms. Recently, the Prf1 pathway has also been associated with different CNS diseases spanning from the eye to the brain, such as choroidal neovascularization in AMD patients, but also in mouse models of experimental stroke [107], TBI [33], Parkinson's [108], and Alzheimer’s diseases [109]. Relevant to our findings, Matsubara et al. [110] demonstrated that retinal degeneration, either due to aging or pathological conditions associated with AMD, is worsened by an increased presence of Prf1 immunoreactive cells in the outer retina.

In conclusion, our data suggest a critical function of Prf1-mediated T-cell neurotoxicity during retinal injury, indicating that targeting infiltrating Prf1^+^ and CD8^+^ T cells may be a potential treatment for retinal degeneration. Specific inhibitors of the Prf1 pathway, already in preclinical studies for non-ocular diseases, may also provide novel strategies to stop these early events associated with the development of retinal degeneration. Nevertheless, future studies are necessary to further research the Prf1 pathway in retinal degenerative diseases. A more detailed characterization of these CD8^+^ T cells is also of interest to determine whether different subsets of CD8^+^ T cells have distinct roles upon retinal injury (e.g., by adoptive transfer of Tc1, Tc2 or Tc17 subsets), which would further complement our results. Thus, understanding these lymphocyte-driven pathological pathways may pave the way for new therapeutic opportunities for retinal degenerative diseases like AMD.

### Supplementary Information

**Additional file 1: Fig. S1. **Validation of T-cell labeling for in vivo imaging. Flow cytometry of the peripheral blood drawn from C57Bl/6 J mice injected retro-orbitally with AF488 anti-CD4 antibody, AF647 anti-CD8 antibody, and AF594 anti-CD3 antibody as an internal control to confirm the correct gating strategy on T cells. We identified lymphocytes, monocytes, and neutrophils by SSC and FSC. We then plotted the SSC of the lymphocyte population against the fluorescent T-cell antibodies. Finally, we re-plotted the result against FSC. We verified that the signal of the antibodies was located where we previously gated the lymphocyte population (purple rings) and concluded that our method efficiently labels the population of interest.

**Additional file 2: Fig. S2.** Assessment of the immune response after anti-CD8 and anti-CD4 treatments. (a) Flow cytometry of the peripheral blood of a Cx3cr1^GFP^Ccr2^RFP^ mouse 24 h before treatment and injury compared to a week after injury (day 7) to monitor the extent of CD4 depletion. The depletion reduced the helper T cells to ~ 1% of the initial population. (b) Flow cytometry of the peripheral blood of a C57Bl/6 J mouse 24 h before treatment and injury compared to two weeks after (day 14) to prove the efficiency of CD8 depletion until the end of the experiment. The depletion reduced the helper T cells to ~ 5% of the initial population.

**Additional file 3: Fig. S3.** Image processing for the quantification of immune responses in pictures obtained by in vivo imaging. The representative image of Cx3cr1^+^ cells shows Cx3cr1^+^ cell shapes (left), including very thin protrusions. After processing and reducing the fluorescent signal (right), the Cx3cr1^+^ cell details are no longer visible, and the cell body is easier to distinguish. The approach for counting Ccr2^+^, CD4^+^, CD8^+^ and Foxp3^+^ cells relies only on ImageJ thresholding. Representative pictures show images before (left) and after (right) the threshold was set.

**Additional file 4: Fig. S4.** Assessment of the immune response after FTY720 treatment. (a-b) Flow cytometry and (c) quantification of B cells (B220^+^/CD3^−^), T cells (B220^−^/CD3^+^) and granulocytes together with macrophages/monocytes (B220^−^/CD3^−^) in the peripheral blood after 3 days of FTY720 treatment. (d) In vivo imaging of the same eye of a C57BL/6 mouse injected with anti-CD45-AF488 to label leukocytes demonstrating that FYT720 treatment prevents with PL recruitment to the injured retinal tissue on days 1, 4 and 7. The damaged area is delimited by magenta dashes. Field of view is ≈425 µm. (e) Images of Cx3cr1^+^ cell morphology at the baseline (before injury) in untreated and FTY720-treated mice show that FTY treatment does not induce cell activation. The arrow indicates the direction in which the damaged site is located. Quantification of the polarization coefficient of untreated and FTY720-treated Cx3cr1^+^ cells. Significant differences between untreated and FTY720-treated mice were determined by using a two-tailed Mann–Whitney test analysis (*n* = 8). (f) Angiographs of untreated and FTY720-treated eyes at the baseline (before injury) demonstrates that FTY720 treatment does not affect blood-retinal barrier integrity.

**Additional file 5: Fig. S5.** Association between Cx3cr1^+^ cell polarization with their clustering during injury response. Spearman's rank-order correlation between the number of Cx3cr1^+^ cells and PL recruited in the injury with polarization coefficient of Cx3cr1^+^ cells in untreated mice. Color intensity and the size of the circle are proportional to the correlation coefficients, and a star (*) marks significant correlations.

**Additional file 6: Fig. S6.** Association between Cx3cr1^+^ cell polarization with PL clustering that is prevented by FTY720 treatment. Spearman's rank-order correlation between the number of Cx3cr1^+^ cells and PL recruited in the injury with polarization coefficient of Cx3cr1^+^ cells in FTY720-treated mice. Color intensity and the size of the circle are proportional to the correlation coefficients, and a star (*) marks significant correlations.

**Additional file 7: Fig. S7.** Association between the clustering of PL with scar formation and BRB damage. Spearman's rank-order correlation between damaged (Injury, top)/leakage area (BRB break, bottom) with the number of Cx3cr1^+^ cells and PL clustering in the injury in untreated mice. Color intensity and the size of the circle are proportional to the correlation coefficients, and a star (*) marks significant correlations.

**Additional file 8: Fig. S8.** Ex vivo analysis of T-cells response to focal injury in the murine retina. The provided images display representative sections stained for CD3 and DAPI, illustrating the cellular composition before injury (uninjured) and 4 days after injury (day 4). These visual observations provide evidence that undamaged tissue lacks T cells, while T cells are present following tissue damage.

**Additional file 9: Fig. S9.** Association between the clustering of CD8^+^ T cells with scar formation and BRB damage. Spearman's rank-order correlation between damaged (Injury, top)/leakage area (BRB break, bottom) with the number of CD4^+^ and CD8^+^ T cells clustering in the injury in untreated mice.

## Data Availability

Not applicable.

## Abbreviations

AF488/594/647: Alexa Fluor 488/594/647
AMD: Age-related macular degeneration
AOM: Acousto-optic modulator
BRB: Blood–retina barrier
CD4/8: Cluster of differentiation 4/8
CW: Continuous-wave
DAPI: 6-Diamidino-2-phenylindole
ERG: Electroretinography
FA: Fluorescein angiography
FOXP3: Forkhead box P3
FTY720: Fingolimod
GFP: Green fluorescent protein
KO: Knockout
ONL: Outer nuclear layer
OPL: Outer plexiform layer
PL: Peripheral leukocytes
PMT: Photomultiplier tube
Prf1: Perforin
RFP: Red fluorescent protein
RPE: Retinal pigment epithelium
S1p1: Sphingosine-1-phosphate receptor 1
SD-OCT: Spectral domain-optical coherence tomography
SLO: Scanning laser ophthalmoscope
TBI: Traumatic brain injury
