# Supplementary figures and images for: Assessing the role of T cells in response to retinal injury to uncover new therapeutic targets for the treatment of retinal degeneration
Source: J Neuroinflammation. 2023 Sep 9;20:206. doi: 10.1186/s12974-023-02867-x (PMC10492418; doi:10.1186/s12974-023-02867-x)

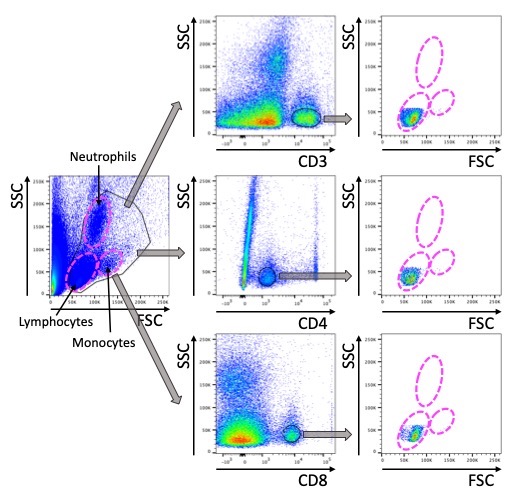

Supplement: Supplementary file 1 — Additional file 1: Fig. S1. Validation of T-cell labeling for in vivo imaging. Flow cytometry of the peripheral blood drawn from C57Bl/6 J mice injected retro-orbitally with AF488 anti-CD4 antibody, AF647 anti-CD8 antibody, and AF594 anti-CD3 antibody as an internal control to confirm the correct gating strategy on T cells. We identified lymphocytes, monocytes, and neutrophils by SSC and FSC. We then plotted the SSC of the lymphocyte population against the fluorescent T-cell antibodies. Finally, we re-plotted the result against FSC. We verified that the signal of the antibodies was located where we previously gated the lymphocyte population (purple rings) and concluded that our method efficiently labels the population of interest. [file 12974_2023_2867_MOESM1_ESM.jpg]

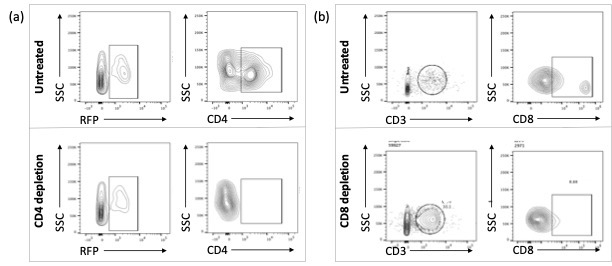

Supplement: Supplementary file 2 — Additional file 2: Fig. S2. Assessment of the immune response after anti-CD8 and anti-CD4 treatments. (a) Flow cytometry of the peripheral blood of a Cx3cr1GFPCcr2RFP mouse 24 h before treatment and injury compared to a week after injury (day 7) to monitor the extent of CD4 depletion. The depletion reduced the helper T cells to ~ 1% of the initial population. (b) Flow cytometry of the peripheral blood of a C57Bl/6 J mouse 24 h before treatment and injury compared to two weeks after (day 14) to prove the efficiency of CD8 depletion until the end of the experiment. The depletion reduced the helper T cells to ~ 5% of the initial population. [file 12974_2023_2867_MOESM2_ESM.jpg]

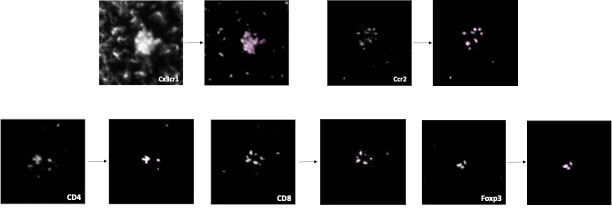

Supplement: Supplementary file 3 — Additional file 3: Fig. S3. Image processing for the quantification of immune responses in pictures obtained by in vivo imaging. The representative image of Cx3cr1+ cells shows Cx3cr1+ cell shapes (left), including very thin protrusions. After processing and reducing the fluorescent signal (right), the Cx3cr1+ cell details are no longer visible, and the cell body is easier to distinguish. The approach for counting Ccr2+, CD4+, CD8+ and Foxp3+ cells relies only on ImageJ thresholding. Representative pictures show images before (left) and after (right) the threshold was set. [file 12974_2023_2867_MOESM3_ESM.jpg]

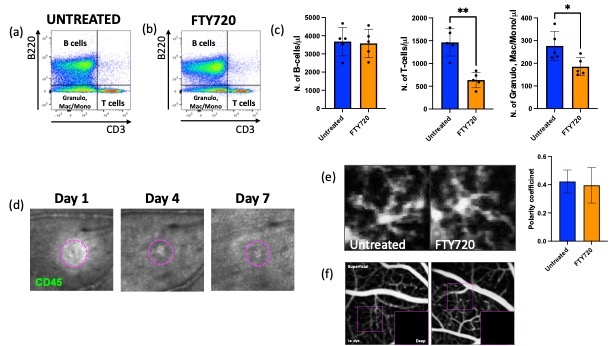

Supplement: Supplementary file 4 — Additional file 4: Fig. S4. Assessment of the immune response after FTY720 treatment. (a-b) Flow cytometry and (c) quantification of B cells (B220+/CD3−), T cells (B220−/CD3+) and granulocytes together with macrophages/monocytes (B220−/CD3−) in the peripheral blood after 3 days of FTY720 treatment. (d) In vivo imaging of the same eye of a C57BL/6 mouse injected with anti-CD45-AF488 to label leukocytes demonstrating that FYT720 treatment prevents with PL recruitment to the injured retinal tissue on days 1, 4 and 7. The damaged area is delimited by magenta dashes. Field of view is ≈425 µm. (e) Images of Cx3cr1+ cell morphology at the baseline (before injury) in untreated and FTY720-treated mice show that FTY treatment does not induce cell activation. The arrow indicates the direction in which the damaged site is located. Quantification of the polarization coefficient of untreated and FTY720-treated Cx3cr1+ cells. Significant differences between untreated and FTY720-treated mice were determined by using a two-tailed Mann–Whitney test analysis (n = 8). (f) Angiographs of untreated and FTY720-treated eyes at the baseline (before injury) demonstrates that FTY720 treatment does not affect blood-retinal barrier integrity. [file 12974_2023_2867_MOESM4_ESM.jpg]

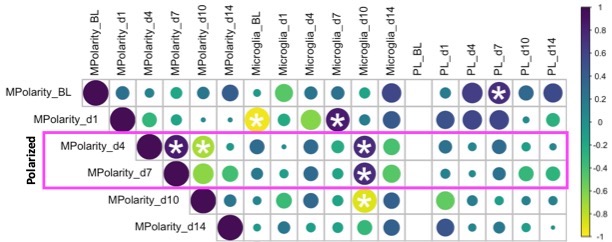

Supplement: Supplementary file 5 — Additional file 5: Fig. S5. Association between Cx3cr1+ cell polarization with their clustering during injury response. Spearman's rank-order correlation between the number of Cx3cr1+ cells and PL recruited in the injury with polarization coefficient of Cx3cr1+ cells in untreated mice. Color intensity and the size of the circle are proportional to the correlation coefficients, and a star (*) marks significant correlations. [file 12974_2023_2867_MOESM5_ESM.jpg]

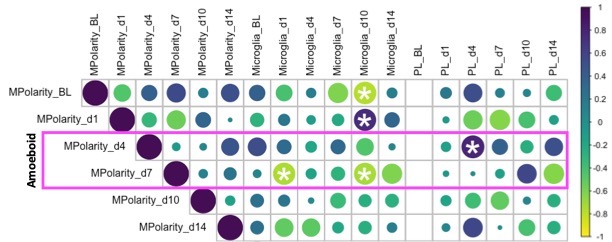

Supplement: Supplementary file 6 — Additional file 6: Fig. S6. Association between Cx3cr1+ cell polarization with PL clustering that is prevented by FTY720 treatment. Spearman's rank-order correlation between the number of Cx3cr1+ cells and PL recruited in the injury with polarization coefficient of Cx3cr1+ cells in FTY720-treated mice. Color intensity and the size of the circle are proportional to the correlation coefficients, and a star (*) marks significant correlations. [file 12974_2023_2867_MOESM6_ESM.jpg]

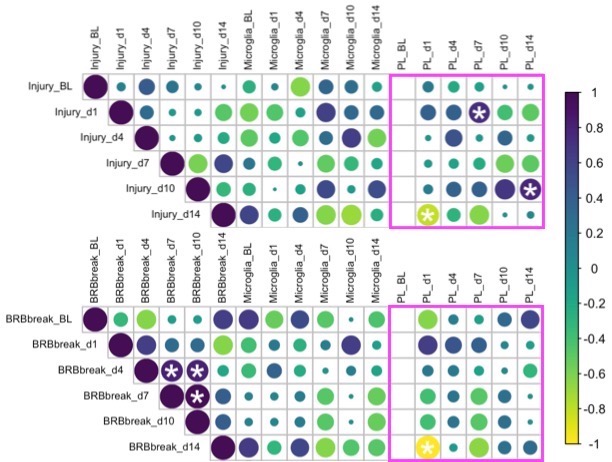

Supplement: Supplementary file 7 — Additional file 7: Fig. S7. Association between the clustering of PL with scar formation and BRB damage. Spearman's rank-order correlation between damaged (Injury, top)/leakage area (BRB break, bottom) with the number of Cx3cr1+ cells and PL clustering in the injury in untreated mice. Color intensity and the size of the circle are proportional to the correlation coefficients, and a star (*) marks significant correlations. [file 12974_2023_2867_MOESM7_ESM.jpg]

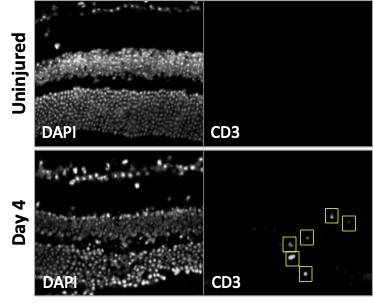

Supplement: Supplementary file 8 — Additional file 8: Fig. S8. Ex vivo analysis of T-cells response to focal injury in the murine retina. The provided images display representative sections stained for CD3 and DAPI, illustrating the cellular composition before injury (uninjured) and 4 days after injury (day 4). These visual observations provide evidence that undamaged tissue lacks T cells, while T cells are present following tissue damage. [file 12974_2023_2867_MOESM8_ESM.jpg]

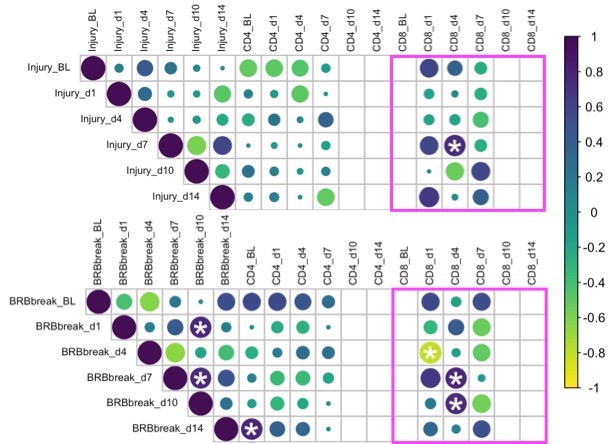

Supplement: Supplementary file 9 — Additional file 9: Fig. S9. Association between the clustering of CD8+ T cells with scar formation and BRB damage. Spearman's rank-order correlation between damaged (Injury, top)/leakage area (BRB break, bottom) with the number of CD4+ and CD8+ T cells clustering in the injury in untreated mice. [file 12974_2023_2867_MOESM9_ESM.jpg]
